# The development and internal pilot trial of a digital physical activity and emotional well-being intervention (Kidney BEAM) for people with chronic kidney disease

**DOI:** 10.1038/s41598-023-50507-4

**Published:** 2024-01-06

**Authors:** Hannah M. L. Young, Ellen M. Castle, Juliet Briggs, Christy Walklin, Roseanne E. Billany, Elham Asgari, Sunil Bhandari, Nicolette Bishop, Kate Bramham, James O. Burton, Jackie Campbell, Joseph Chilcot, Nicola Cooper, Vashist Deelchand, Matthew P. M. Graham-Brown, Lynda Haggis, Alexander Hamilton, Mark Jesky, Philip A. Kalra, Pelagia Koufaki, Jamie Macdonald, Kieran McCafferty, Andrew C. Nixon, Helen Noble, Zoe L. Saynor, Maarten W. Taal, James Tollitt, David C. Wheeler, Thomas J. Wilkinson, Sharlene A. Greenwood

**Affiliations:** 1 Leicester Diabetes Centre, University Hospitals of Leicester NHS Trust, Leicester, UK; 2 Diabetes Research Centre, University of Leicester, Leicester, UK; 3 National Institute of Health Research Leicester Biomedical Research Centre, Leicester, UK; 4 School of Physiotherapy, Department of Health Sciences, Brunel University, London, UK; 5 Renal Department, King’s College Hospital, London, UK; 6 Department of Cardiovascular Sciences, University of Leicester, Leicester, UK; 7 Department of Nephrology, Guys and St Thomas’s Hospital, London, UK; 8 Department of Nephrology, Hull University Teaching Hospitals NHS Trust, Hull, UK; 9 School of Sport, Exercise and Health Sciences, Loughborough University, Loughborough, UK; 10 Faculty of Health, Education and Society, University of Northampton, Northampton, UK; 11 Institute of Psychiatry, Psychology & Neuroscience, King’s College London, London, UK; 12 Department of Population Health Science, University of Leicester, Leicester, UK; 13 Department of Nephrology Royal Free Hospital, London, UK; 14 Department of Nephrology, Royal Devon and Exeter NHS Foundation Trust, Exeter, UK; 15 Department of Nephrology, Nottingham NHS Trust, Nottingham, UK; 16 Department of Nephrology Royal Hospital, Northern Care Alliance NHS Foundation Trust, Salford, UK; 17 Dietetics, Nutrition & Biological Sciences, Physiotherapy, Podiatry & Radiography Division, Queen Margaret University, Edinburgh, UK; 18 Institute for Applied Human Physiology, Bangor University, Bangor, UK; 19 Department of Nephrology, Barts Health NHS Trust, London, UK; 20 Department of Renal Medicine, Lancashire Teaching Hospitals NHS Foundation Trust, Preston, UK; 21 Division of Cardiovascular Sciences, The University of Manchester, Manchester, UK; 22 School of Nursing and Midwifery, Queen’s University Belfast, Belfast, UK; 23 School of Sport, Health and Exercise Science, University of Portsmouth, Portsmouth, UK; 24 Centre for Kidney Research and Innovation, University of Nottingham, Nottingham, UK; 25 Department of Renal Medicine, University College London, London, UK

**Keywords:** Nephrology, Quality of life

## Abstract

This trial assessed the feasibility and acceptability of Kidney BEAM, a physical activity and emotional well-being self-management digital health intervention (DHI) for people with chronic kidney disease (CKD), which offers live and on-demand physical activity sessions, educational blogs and videos, and peer support. In this mixed-methods, multicentre randomised waitlist-controlled internal pilot, adults with established CKD were recruited from five NHS hospitals and randomised 1:1 to Kidney BEAM or waitlist control. Feasibility outcomes were based upon a priori progression criteria. Acceptability was primarily explored via individual semi-structured interviews (*n* = 15). Of 763 individuals screened, *n* = 519 (68%, 95% CI 65 to 71%) were eligible. Of those eligible, *n* = 303 (58%, 95% CI 54–63%) did not respond to an invitation to participate by the end of the pilot period. Of the 216 responders, 50 (23%, 95% CI 18–29%) consented. Of the 42 randomised, *n* = 22 (10 (45%) male; 49 ± 16 years; 14 (64%) White British) were allocated to Kidney BEAM and *n* = 20 (12 (55%) male; 56 ± 11 years; 15 (68%) White British) to the waitlist control group. Overall, *n* = 15 (30%, 95% CI 18–45%) withdrew during the pilot phase. Participants completed a median of 14 (IQR 5–21) sessions. At baseline, 90–100% of outcome data (patient reported outcome measures and a remotely conducted physical function test) were completed and 62–83% completed at 12 weeks follow-up. Interview data revealed that remote trial procedures were acceptable. Participants’ reported that Kidney BEAM increased their opportunity and motivation to be physically active, however, lack of time remained an ongoing barrier to engagement with the DHI. An randomised controlled trial of Kidney BEAM is feasible and acceptable, with adaptations to increase recruitment, retention and engagement.

**Trial registration** NCT04872933. Date of first registration 05/05/2021.

## Introduction

Chronic kidney disease (CKD) affects over 800 million adults worldwide and is associated with lower health-related quality of life (HRQoL)^1,2^. Physical activity and emotional- wellbeing self-management support have a beneficial impact on HRQoL in this population^3^, and are key elements in the management of many long-term conditions^4^. Despite this, there is limited access to this type of care for people with CKD^4,5^.

Digital health interventions (DHIs) may offer a clinically and cost-effective means of addressing barriers to accessing support for physical activity and emotional well-being^6–8^. Digital health interventions can significantly improve clinical outcomes in people with CKD, but existing DHIs do not specifically focused upon physical activity and emotional well-being^4,9^. There is an urgent need for high-quality, large-scale trials focussing upon patient-centred outcomes^9^. In addition, reviews of barriers and facilitators to physical activity DHIs suggest that they need to be tailored to the requirements of the specific long-term condition^10^, yet here is little research into the specific views of people with CKD concerning DHIs^9,11^. Understanding these perspectives and beliefs is crucial to ensuring that DHIs meet the needs of a diverse range of people^9^.

### Aims

Kidney BEAM is a DHI which has been co-developed with people with CKD to support physical activity and emotional well-being self-management. A multicentre prospective, wait-list randomised controlled trial (RCT) will evaluate whether Kidney BEAM improves HRQoL^12^. The aims of this trial’s internal pilot were to (1) establish the feasibility of a RCT investigating the effectiveness of Kidney BEAM and (2) explore participants’ perceptions of trial and intervention acceptability. This paper describes the development of Kidney BEAM, and the methods and results of the internal pilot.

## Methods

Reporting is informed by relevant CONSORT extensions^13–15^ and reporting guidance for remote trials^16^ and qualitative research^17^.

### Design

A prospective, mixed-methods, multicentre randomised waitlist-controlled internal pilot trial was conducted. An internal pilot is a phase in a trial after which progress is assessed against pre-specified progression criteria^18^. Internal pilots are a cost-effective way of assessing whether it is feasible to continue to a definitive trial, as data collected during the internal pilot phase contribute towards the final analyses^18^. The results may allow trialists to modify the processes used to enhance the chances of successful recruitment and retention, alongside other important feasibility parameters^13^. All procedures were performed in accordance with the relevant guidelines and regulations. The trial was prospectively registered (NCT04872933). Date of first registration 05/05/2021.

### Settings and participants

Adults > 18 with established CKD were recruited from five NHS hospitals within the UK. Centres selected were those first open to recruitment. Inclusion and exclusion criteria mirrored the definitive trial (Table 1)^12^. CKD was defined as kidney damage or glomerular filtration rate (GFR) < 60 mL/min/1.73 m(2) for 3 months or more, irrespective of cause^19^.

**Table 1:** Inclusion and exclusion criteria for the Kidney BEAM pilot trial.

Inclusion criteria	Exclusion criteria
Individuals with established CKD of all stages, including those undergoing renal replacement therapy (haemodialysis, peritoneal dialysis and kidney transplant)	Weight < 50 kg
Adults aged 18 years or older	Self-reported participation in a structured exercise programme or kidney BEAM digital health intervention within the previous 3 months
Access to a Wi-Fi-enabled device	Active infection
Able to understand written English language	Uncontrolled arrhythmias
Able to provide written or virtual informed consent	Unstable angina or heart attack within 3 months
	Persistent uncontrolled hypertension (systolic blood pressure > 180 mm Hg or diastolic blood pressure > 110 mm Hg)
	Recent (within the last 3 months) stroke or transient ischaemic attack
	Receiving palliative care for advanced or terminal cancer
	Adults with peripheral vascular or musculoskeletal disease, who the Principal Investigator deemed unable to carry out a physical activity intervention
	Any other health condition considered by the local Principal Investigator in which physical activity would be contraindicated
	Insufficient understanding of the trial

### Recruitment

Routine clinical staff who were engaged in delivering routine care at each hospital site screened medical records to identify eligible participants. Suitable adults were primarily approached via telephone, or face-to-face during routine clinic visits, by trained research staff. Posters were also displayed at each kidney unit, allowing potential participants to contact the trial team if they were interested. Written informed consent was obtained primarily via an online link or face-to-face at a routine clinic visits.

### Baseline demographic and clinical variables

In addition to the outcome measures collected (see below), demographic and clinical characteristics were gathered from participant records.

### Randomisation

Following baseline assessments, participants were randomised (1:1) to Kidney BEAM or the waitlist control group, using randomly permuted blocks. Randomisation was performed by an independent researcher using a validated web-based system (Sealed Envelope Ltd).

### Wait list group

Participants who were allocated to the wait-list control group continued with their usual care and refrained from participating in a structured exercise programme. They were invited to use Kidney BEAM following the end of their involvement in the trial.

### Overview of the development of the Kidney BEAM intervention

#### Initial development phase

The development process, and intervention logic model, are detailed in Supplementary Material 1. Development was informed by INDEX, DHI and co-production guidance^20–23^. Fifteen researchers and clinicians with backgrounds in rehabilitation, physical activity, nephrology and digital health, five web developers, six partners from kidney charities (Supplementary Material 1) and six stakeholders with expertise through lived experience of CKD (50% male; 53 ± 17 years; 50% White British; 33% pre-dialysis CKD, 33% dialysis, 33% transplanted) co-produced the intervention. Stakeholders with lived experience volunteered for this role and were engaged via Kidney Research UK networks, or via the hospital sites involved in the trial.

The initial theory-informed development of Kidney BEAM included four iterative stages, which occurred rapidly over four-weeks:

1. Existing evidence and ongoing research were reviewed and matched to the COM-B model within a behavioural analysis^24–29^.
2. Potential levers for change, identified by the behavioural analysis, were linked with intervention functions using the Behaviour Change Wheel^30^. The affordability, practicality, effectiveness, acceptability, safety, equity (APEASE)^29^ and contextual fit^20^ of the proposed functions were considered.
3. Identified functions were linked to appropriate behaviour change techniques using the behaviour change taxonomy^31^.
4. Participatory co-design^23^ and user-centred agile software design^32^ were used to create an intuitive and engaging DHI.

#### User testing phase

A six-month user testing phase to direct further refinement followed development. This is reported elsewhere^7^ and summarised in Supplementary Material 1. The final intervention is described in detail in Table 2^33^. Briefly, Kidney BEAM is a digital health intervention (DHI) co-developed with people with CKD to support physical activity and emotional well-being self-management. Kidney BEAM can be accessed via a range of devices (including smartphones) and offers: online live exercise classes modelled on kidney rehabilitation programmes led by a kidney physiotherapist; on-demand exercise videos offering a range of different types of physical activity of varying durations and for different levels of fitness and ability; educational blogs and videos on a variety of topics relating to CKD self-management; virtual groups for peer support; a personalised schedule for booking live classes, organising activities, setting personal goals, and reminders and; a personalised physical activity diary to monitor progress and record off-DHI activity. The materials on BEAM were provided by a wide range of kidney professionals and qualified exercise instructors who themselves were living with CKD.

**Table 2:** Template for intervention description and replication checklist for the Kidney BEAM digital health intervention.

Description	Kidney BEAM is a digital health intervention (DHI) co-developed with people with CKD to support physical activity and emotional well-being self-management. Kidney BEAM can be accessed at https://beamfeelgood.com/home	
Rationale	There is strong evidence for the benefits of physical activity in the CKD population but limited access to such support within routine practice. In addition, people with CKD report a variety of barriers to becoming more active, which have been further compounded by the coronavirus-19 pandemic. DHIs are widely accessible and have the potential to address these barriers, but to date, there appear to be no existing DHIs that provide physical activity or emotional well-being support specifically for people with CKD	
What	Materials provided to participants	Kidney BEAM can be accessed via a range of devices (including smartphones) and offers: • Online live exercise classes modelled on kidney rehabilitation programmes • On-demand exercise videos, offering a range of different types of physical activity (e.g. yoga, Pilates, high-intensity interval training), of varying durations (10–60 min) and for different levels of fitness and ability (beginner, intermediate and advanced) • Educational blogs and videos on a variety of topics relating to CKD self-management (e.g. managing your blood pressure), physical activity in CKD (e.g. the benefits of strength training, staying steady) • Virtual groups for peer support • Personalised schedule for booking live classes and organising activities and setting personal goals, and reminders • Personalised physical activity diary to monitor progress against recommended weekly physical activity targets, engagement with Kidney BEAM and record off-DHI activity • Engagement with the website is encouraged through regular email updates, action planning, self-monitoring of behaviour, prompts/cues, and newsletters
	Materials used to train intervention providers	• All instructors/intervention providers involved in the delivery of classes and the creation of recorded educational videos and blogs were specialist physiotherapists or exercise instructors with extensive experience in the delivery of physical activity support to this population or qualified exercise instructors who themselves were living with CKD • Additional educational content was also provided by nephrologists and kidney counsellors
	Procedures, activities, and/or processes used in the intervention	• Creating a user profile for participants • Undertaking safety assessments before participation in live classes • Monitoring adherence and providing telephone and email-based support and reminders to participants struggling to engage with Kidney BEAM • Provide live exercise and education classes modelled on renal rehabilitation and recorded on-demand classes and education • Provide feedback on exercise technique and progression to those who request further support within the live class settings
Who (intervention providers)	The materials on BEAM were provided by kidney professionals, including: • Kidney specialist physiotherapists • Nephrologists • Kidney counsellors • Researchers with expertise in the field of physical activity and CKD from several different NHS Trusts and higher education institutes, and qualified exercise instructors who themselves were living with CKD Live exercise classes led by a kidney physiotherapist from the lead site (Kings College Hospital NHS Trust) and a Physiotherapy Technical Instructor. These providers were delivering the sessions as part of their clinical role. No incentives were provided	
How (mode of delivery)	Kidney BEAM was delivered online and accessible using a range of devices and operating systems. Engagement was encouraged via email notification and telephone follow-ups dependent on adherence	
Where (location)	Participants were able to use Kidney BEAM in a variety of settings. Most undertook the on-demand and live classes at home via their smartphone, tablet or PC The Kidney BEAM pilot evaluation was undertaken in five NHS centres in the UK: Kings College Hospital London, University Hospitals of Leicester NHS Trust, Portsmouth Hospitals NHS Trust, University Hospitals of Derby and Burton NHS Foundation Trust, and Barts Health NHS Trust. These are all large NHS kidney centres, serving a socioeconomically and ethnically diverse population	
When and how much	The frequency of delivery	Participants were encouraged to attend a minimum of twice weekly physical activity sessions, to achieve 150 min/per week of moderate-intensity aerobic activity or 75 min/per week of vigorous activity, and muscle resistance training on 2 days of the week
	Target intensity	Participants were coached on how to use Borgs rating of perceived exertion to rate the intensity of their sessions. Moderate-intensity exercise was achieved at a Rating of Perceived Exertion (RPE) of 12–14
	Target duration	The duration of the live exercise classes was ~ 40 min and the duration of the on-demand physical activity sessions ranged from 10 to 40 min. The on-demand renal rehab programme followed the same target duration of ~ 40 min as per live classes
	The total duration of delivery	12 weeks. At the end of the 12-week programme, participants were encouraged by phone and email to maintain their engagement and self-manage their activity via kidney BEAM
Tailoring	Participants were able to select a range of physical activity/exercise types that suited their needs and interests. Live exercise classes were tailored based on participants’ functional abilities and progressed using Borgs rating of perceived exertion In addition, tailored support to use Kidney BEAM was provided to those who reported challenges with digital literacy, including: • Telephone support, for people requiring technical help • An arrangement with Citizens Online, a digital inclusion charity that provides access to in-person and telephone support with digital champions who provide skills training and can support access to Kidney BEAM • ‘How to’ guides and video demonstrations that can be shared via email • Prompts and trouble-shooting with individuals if adherence is reduced, for example when unwell	

Participants were encouraged to attend a minimum of twice weekly physical activity sessions, to achieve 150 min/per week of moderate-intensity aerobic activity or 75 min/per week of vigorous activity, and muscle resistance training on 2 days of the week. All participants had access to the DHI for 12 weeks. At the end of the 12-week programme, participants were encouraged by phone and email to maintain their engagement and self-manage their activity using the DHI.

### Sample size

Determinations of sample size around a primary outcome are not relevant to an internal pilot, and sample sizes of 24–50 are considered sufficient^34^. For the qualitative component, maximum variation sampling was used to ensure diversity^35^. Primary importance was given to sampling participants with a diverse range of engagement with Kidney BEAM. Fifteen interviews were conducted, with data collection ceasing when information power was sufficient to address the research aims^36^.

### Primary outcome for the internal pilot

#### Eligibility and recruitment

The feasibility of remote recruitment was established by recording rates of eligibility and recruitment. The demographics of those who declined were also recorded.

#### Randomisation and baseline assessments

The acceptability of randomisation and remote assessment procedures were explored by examining the proportion of participants withdrawing at each time point by group.

#### Intervention acceptability

To explore the acceptability of Kidney BEAM, in addition to the qualitative interviews described below, adherence was assessed using the number of sessions (live and on-demand) completed, minutes of physical activity undertaken and the proportion of participants who completed at least one (a key progression criterion) and two (the minimum recommended number) sessions per week over 12 weeks.

#### Outcome acceptability

Rates of outcome completion were used to determine the ability to collect data remotely at baseline and 12 weeks. Patient reported outcome measures (PROMs) were gathered using a secure online questionnaire (SurveyMonkey Ltd) (Table 3). Participants also remotely undertook the Sit-to-Stand in 60 Seconds (STS60), a validated measure of muscle endurance^37^. Blinding of the intervention providers and the participants was not possible. Outcome assessors were, however, blinded to treatment allocation.

**Table 3:** Secondary outcomes included within the pilot trial.

Measure	Description
Kidney disease quality of life (KDQoL SF1.3)	Health-related quality of life
EQ-5D-5L	
The Chalder fatigue questionnaire	Physical and mental fatigue
The work and social adjustment scale (WSAS)	Life participation
Patient activation measure (PAM-13)	Patient activation (the knowledge, skills and confidence a person has in managing their own health and health care)
Global physical activity questionnaire (GPAQ)	Self-reported physical activity
Patient-health questionnaire-4 (PHQ-4)	Depression and anxiety

#### Safety and monitoring

All adverse events (AEs) and serious adverse events (SAEs) were recorded and considered for severity, causality and expectedness.

#### Assessment of feasibility

For key feasibility outcomes, a priori criteria (Table 4) were used to establish progression to a definitive trial^38^. For each criterion, ‘stop’ (indicating there were fundamental challenges that may impede a definitive trial) and ‘go’ thresholds (indicating there were no challenges) were established. Results falling between these thresholds indicated that adaptations may render the definitive trial viable^38^.

**Table 4:** Progression criteria for the Kidney BEAM pilot trial.

Recruitment Based on 8 centres, aiming to recruit an overall total of *n* = 304 participants within 9 months	Stop	Three centres are unable to open to recruitment with the pilot period
	Amber	Less than *n* = 15 eligible participants are recruited per centre, within 12 weeks but other centres can make up the shortfall
	Go	*n* = 15 participants recruited within 12 weeks per centre
Intervention acceptability	Stop	Engagement with at least one BEAM class per week in 50% of participants or less
	Amber	Engagement with at least one BEAM class per week for 51–69% of participants
	Go	Engagement with at least one BEAM class per week for at least 70% of participants
Outcome acceptability Based upon completion of the KDQoL SF1.3	Stop	Less than 60% completion rate
	Amber	61–79% completion rate
	Go	At least 80% completion rate
Loss to follow up	Stop	More than 50% dropout rate during the pilot phase
	Amber	A dropout rate of 21–49%
	Go	Less the 20% dropout rate

### Interviews

One-to-one, semi-structured interviews with participants from the intervention group explored their: (i) perceptions of Kidney BEAM and; (ii) experiences of participating in a remote trial. A topic guide (Supplementary Material 2) was developed in advance and piloted during the first three interviews. These initial interviews were retained within the analysis.

Interviews were conducted by experienced qualitative researchers, all of whom had experience of working with people with CKD (HMLY, EMC, RB, JB). Some of the participants may have been aware of the researchers from their role in developing and delivering physical activity and education as part of the DHI. Any influence of these dual roles on the data collection or analysis process was explored via reflexive journals kept by the qualitative research team throughout, creating an ‘audit trail’ of analytical decisions. The involvement of multiple researchers in the analysis and interpretation of the data facilitated discussion on the level of agreement between coders and ensured that analysis and interpretation remained grounded in the data. To enhance credibility, the findings were discussed with the wider multidisciplinary research team and the patient and public (PPI) group. Interviews were conducted at an appointment separate from the trial assessments via telephone or secure online video conferencing software and were audio-recorded. Participants were interviewed as soon as possible following the completion of their involvement in the trial.

### Data analysis

Quantitative analyses were performed using SPSS 28 (IBM UK Ltd, UK). Descriptive statistics were used to estimate feasibility outcomes and summarise adverse events^13^ and are presented as mean ± standard deviation, median (IQR) or *n* (%), as appropriate.

Qualitative analysis was undertaken according to the framework approach^39^. Transcripts were reviewed and coded line by line. Initial codes were discussed and refined, culminating in an analytical framework that was then systematically applied to the remaining transcripts. A matrix, which summarised the data from each participant by theme, was created. All qualitative researchers kept a diary to enhance reflexivity and rigour^39,40^. Data management was facilitated using NVivo (QSR International Ltd, version 20) and Excel software (Microsoft Ltd).

Finally, qualitative and quantitative results were merged in a ‘joint display’ to facilitate a comprehensive overall assessment of feasibility and acceptability^41^. This was used alongside the progression criteria to inform progression to a definitive trial. The suggested adaptations to the intervention were prioritised using the MoSCoW framework, which is a feature of agile software design^32^. Prioritisation was informed by the APEASE criteria (Supplementary Material 1)^29^.

### Patient and public involvement (PPI)

The PPI group involved in development of the intervention were also involved in the conception of the trial, and throughout its conduct.

### Ethics approval and consent to participate

The internal pilot was part of the definitive kidney BEAM trial and received ethical approval from the UK National Health Service (NHS) Research Ethics Committee (REC reference 21/LO/0243). All participants provided written informed consent to participate.

## Results

### Feasibility trial

#### Eligibility and recruitment

**Figure 1:**
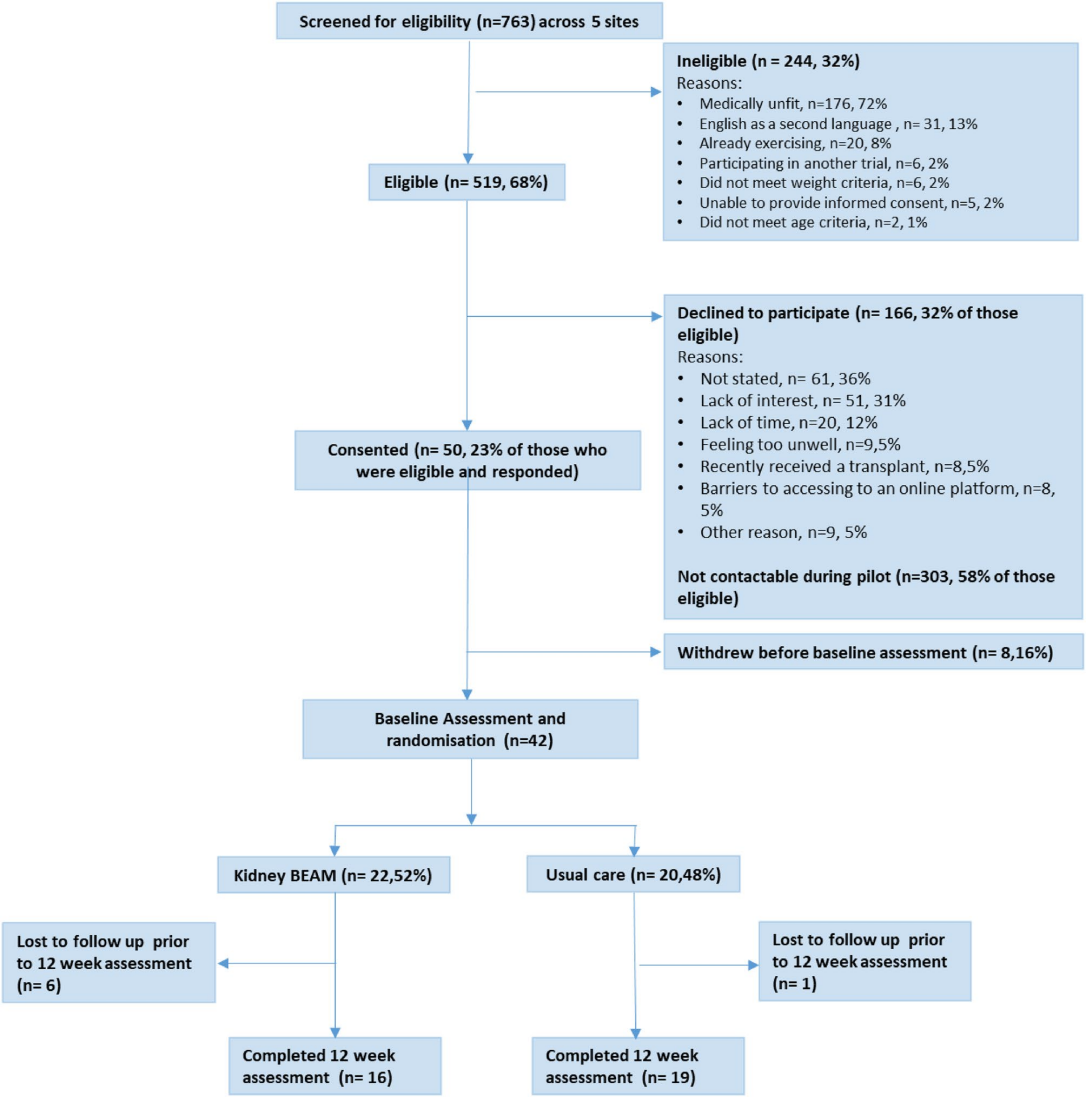
CONSORT flow diagram depicting the progression of participants through the pilot trial.

Screening and recruitment occured from May to December 2021, with data collection completed by June 2022. Figure 1 outlines the progression of participants through the pilot trial. Of the 763 adults screened, *n* = 244 (32%, 95% CI 29 to 35%) were ineligible.

*N* = 519 (68%, 95% CI 65 to 71%) participants were eligible, but *n* = 303 (58%, 95% CI 54–63%) did not respond to an invitation to participate by the end of the pilot period. Of the 216 responders, 50 (23%, 95% CI 18–29%) consented. The majority of those who declined did not provide a reason (*n* = 61, 36%, Fig. 1). Those who declined were similar in demographic to those who participated (Supplementary Material 3).

**Figure 2:**
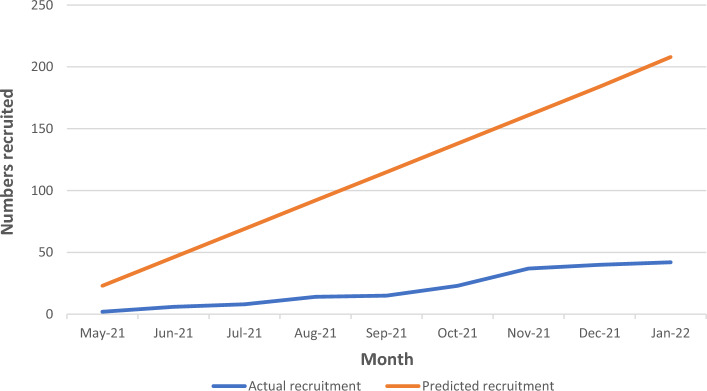
Projected and actual rates of recruitment during the pilot trial.

Cumulative monthly recruitment rates are compared with predicted rates in Fig. 2. Sites recruited a median of 6 (IQR 3–13) participants per month. Of those who consented, *n* = 8 (16% 95% CI 7–29%) withdrew before randomisation. All withdrew through choice or because they did not attend the baseline remote assessment. Of the *n* = 42 randomised, *n* = 22 were allocated to Kidney BEAM and *n* = 20 to the waitlist control group.

#### Participant characteristics

At baseline the groups were well-matched, although the control group were older and had more co-morbidity, as assessed using the Charlson co-morbidity index (Table 5). The majority of the sample were White British (*n* = 29, 69%), and non-frail, as assessed using the Clinical Frailty Scale(*n* = 42, 100%).

**Table 5:** Baseline demographic and clinical characteristics of the trial participants.

		Waitlist control (n = 20)	Kidney BEAM (n = 22)	All (n = 42)
Age (years)		56 ± 11	49 $\pm$ 16	52 $\pm 14$
Sex (*n*, %)	Female	12 (60%)	12 (45%)	20 (48%)
	Male	8 (40%)	10 (55%)	22 (52%)
Ethnicity (*n*, %)	White	15 (75%)	14 (64%)	29 (69%)
	Asian or Asian British	1 (5%)	2 (9%)	3 (6%)
	Black or Black British	1 (5%)	4 (19%)	5 (12%)
	Chinese	1 (5%)	1 (5%)	2 (5%)
	Other ethnic group	1 (5%)	0 (0%)	1 (2%)
	Not stated	1 (5%)	1 (5%)	2 (5%)
Diagnosis (*n*, %)	Glomerular disease	6 (30%)	4 (18%)	10 (24%)
	Diabetic nephropathy	3 (15%)	0 (0%)	3 (7%)
	Tubulointerstitial nephritis	0 (0%)	1 (5%)	1 (2%)
	Hypertension/renal vascular disease	1 (5%)	4 (18%)	5 (12%)
	Familal/heriterdary nephropathies	5 (25%)	8 (36%)	13 (31%)
	Other systemic diseases	1 (5%)	0 (0%)	1 (2%)
	Miscellaneous diseases	4 (20%)	5 (23%)	9 (21%)
Charlson comorbidity index*		3 (3–4)	2 (2–3)	3 (2–4)
CKD stage (*n,* %)	Stage 1	2 (10%)	3 (14%)	5 (12%)
	Stage 2	0 (0%)	1 (5%)	1 (2%)
	Stage 3a	0 (0%)	1 (5%)	1 (2%)
	Stage 3b	1 (5%)	1 (5%)	2 (5%)
	Stage 4	2 (10%)	2 (9%)	4 (9%)
	Stage 5	5 (25%)	3 (14%)	8 (19%)
	Haemodialysis (home or in-centre)	3 (15%)	6 (27%)	9 (21%)
	Peritoneal dialysis	0 (0%)	2 (9%)	2 (5%)
	Transplant	7 (35%)	3 (14%)	10 (24%)
Clinical frailty scale score	1 – very fit	1 (5%)	5 (23%)	6 (14%)
	2 – fit	6 (30%)	7 (32%)	13 (31%)
	3 – managing well	10 (50%)	7 (32%)	17 (40%)
	4 – vulnerable	3 (15%)	3 (14%)	6 (14%)
	5 – mildly frail	0 (0%)	0 (0%)	0 (0%)
	6 – moderately frail	0 (0%)	0 (0%)	0 (0%)
	7 – severely frail	0 (0%)	0 (0%)	0 (0%)
	8 – very severely frail	0 (0%)	0 (0%)	0 (0%)
Total no. medications		10 $\pm$ 4	7 $\pm$ 3	9 $\pm 3$
Clinical information	Creatinine (g/L)*^+^	158 (107–248)	187 (93–459)	162 (105–388)
	eGFR (g/L)*^+^	37 (20–49)	27 (10–62)	32 (13–60)
	Hb (g/L)	122 $\pm$ 17	119 $\pm 22$	120 $\pm$ 19
	SBP (mmHg)*	129 (119–137)	131 (122–140)	129 (121–137)
	DBP (mmHg)*	77 (67–86)	75 (68–84)	76 (68–85)
	BMI	27.12 $\pm 5.23$	26.17 $\pm 5.04$	26.72

Values reported are mean and SD ( ±), except for *median and IQR.

*BMI* body mass index, *CCI* Charlson comorbidity index, *CKD* chronic kidney disease, *DBP* diastolic blood pressure, *SBP* systolic blood pressure.

^+^Data provided only for non-dialysis dependent participants and those in receipt of a transplant.

#### Retention

Seven participants (17%, 95% CI 7–31%) were lost to follow-up; six (14%, 95% CI 5–28%) from the kidney BEAM group and one (2%, 95% CI 0.6–1%) from the waitlist control group. Reasons for withdrawal included kidney transplantation (*n* = 3), being medically unfit (*n* = 2) and personal reasons (*n* = 2).

#### Exercise adherence

Amongst the remaining participants, a median of 14 (IQR 5–21) sessions were completed, representing a median adherence rate of 58% (IQR 28–86) and a median of 729 (IQR 412–1010) minutes of physical activity over 12 weeks, the equivalent of 61 min per week.Thirteen (59%) participants completed at least 1 session.

#### Outcome acceptability

At baseline, 90–100% of outcome data (including the primary outcome for the definitive trial) were completed and 62–83% at 12 weeks (Table 6).

**Table 6:** Secondary outcome measure completion rates at baseline and 12 weeks.

Measure	Proportion completed at baseline n (%)	Proportion completed at 12 weeks n (%)
Kidney disease quality of life (KDQoL SF1.3)	42 (100%)	35 (83%)
EQ-5D-5L	42 (100%)	35 (83%)
The Chalder fatigue questionnaire	42 (100%)	34 (81%)
The work and social adjustment scale (WSAS)	42 (100%)	34 (81%)
PAM-13	42 (100%)	35 (83%)
Global physical activity questionnaire (GPAQ)	39 (90%)	26 (62%)
Patient-health questionnaire-4 (PHQ-4)	42 (100%)	34 (81%)
Sit to stand 60 (STS60)	42 (100%)	35 (83%)

#### Harms

There were no adverse events, serious adverse events or deaths during the pilot.

### Qualitative findings

Twenty participants were approached for an interview and fifteen (75%) agreed. Full characteristics are provided in Table 7. Interviews lasted a median of 43 ± 14 min.

**Table 7:** Demographic and clinical characteristics of the qualitative participants.

		N = 15
Age (years)*		50 (35–58)
Sex *n* (%)	Female	8 (53%)
	Male	7 (47%)
Ethnicity *n* (%)	White British	11 (73%)
	Asian or Asian British	2 (13%)
	Black or Black British	1 (7%)
	Mixed background	1 (7%)
Diagnosis* n* (%)	Glomerular disease	4 (27%)
	Diabetic nephropathy	0 (0%)
	Tubulointerstitial nephritis	1 (7%)
	Hypertension/ renal vascular disease	3 (20%)
	Familal/heriterdary nephropathies	3 (20%)
	Other systemic diseases	0 (0%)
	Miscellaneous diseases	4 (27%)
CKD stage *n* (%)	1	3 (20%)
	2	1 (7%)
	3a	2 (13%)
	3b	0 (0%)
	4	1 (7%)
	5	1 (7%)
	Haemodialysis	2 (13%)
	Peritoneal dialysis	2 (13%)
	Transplant	3 (20%)
Duration of transplant in months* (*n* = 3 participants)		203 (162–234)
Time of dialysis in months* (*n* = 4 participants)		12 (6–15)
Number of Kidney BEAM sessions completed*		14 (12–23)
Adherence categories *n *(%)	Very high (more than 24 sessions)	2 (13%)
	High (18–24 sessions, 75–100% adherence)	4 (27%)
	Moderate (12–17 sessions, 50–74%)	6 (40%)
	Low (less than 12 sessions, less than 50%)	3 (20%)

Values reported are mean and SD ( ±), except for *median and IQR.

*CKD* chronic kidney disease.

### Perceptions of participating in a remote trial

#### Reasons for participation

Illustrative quotes are presented in Table 8. The majority of participants participated due to a pre-existing desire to be active. Many had previously been active, but found this was negatively impacted by lockdown, and Kidney BEAM was a useful ‘kick-starter’ to addressing this. Participants with low to moderate adherence described how a desire to help others and a feeling of indebtedness to their clinical care team were strong drivers to participation.

**Table 8:** Illustrative quotations relating to participants’ perceptions of Kidney BEAM.

Reasons for participation	Desire to be physically active *“We were coming out of lockdown and getting out again and because of being inside for so long, I didn’t exercise as much anymore and I…lost my strength. I found it more difficult to exercise and to walk around, so I thought oh I could take part in this and it’ll…help me get back on track again.”* (Female, teens, Asian British, peritoneal dialysis, moderate adherence) *“I really wanted to improve my fitness, obviously it’s very easy when you have kidney failure because you feel crappy all the time, to not do much, but I do think long term that does make you feel worse and it’s much better to sort of push yourself to keep active”* (Female, 20 s, White British, haemodialysis, moderate adherence)
	Altruism *“I'm always interested in kind of taking part in research with kidney failure. I've done a couple of other ones, quite different, but it’s always I think having more information about something is better and, you know, BEAM’s trying to help people, so if I can give my feedback and experiences, I don't know if they would be helpful, but maybe it would.”* (Male, 50 s, White British, CKD stage 4, moderate adherence) *“I was asked by the…consultant and I have a lot of time for him, his care and his team’s care has been very good – and because he’s asked I said ‘well yes of course.’”* (Female, 50 s, White British, haemodialysis, low adherence)
Experiences of remote recruitment and assessment	Remote recruitment and consent *“[The recruitment process]* was very straightforward, to be honest with you. Basically, I just filled out a form and then I was told [the research team] will get in touch with you…that was all virtual you know. Super easy.” (Male, 30 s, Asian British, kidney transplant, moderate adherence) ***“****I mean, signing up…it kind of made it a bit quick and easier. I didn’t really have to do much… so it was good in that way. I think I got a phone call which explained it all, called me at that time, but I think a phone call detailing what the study was, what the aims of the study were and so forth and that sort of clarified things, I mean, obviously all the written documentation was provided too, but I’d say specifically the phone call was great because then you're able to ask questions”* (Male, 40 s, White British, stage 3a, low adherence)
	Encouraging participation ***“****I think personal stories, you know, get a few people coming on and saying ‘Oh yeah, I joined such and such a class, it’s made a big difference… nice community I joined, this is exercise that anybody can do’ type of thing, yeah, personal experiences I think always help people make a decision.”* (Female, 60 s, mixed background, CKD stage 3a, very high adherence) *“Maybe having something in there, sort of one of the things from someone that’s done it saying “I done this. It’s helped me with this” so maybe that bit of – you know, the information you sent out is fine; maybe having something where even like something has a little video, or something just from someone that’s done it and explaining how it does – a bit more encouragement.”* (Male, 50 s, White British, kidney transplant, moderate adherence)
	Randomisation *“I was glad that I got into it straight away. I think I was sort of curious as to why that was. I didn’t quite understand why, I think potentially I may have thought if I didn’t do it the first time, that I hadn’t qualified for some reason and it might make me wonder why hadn’t I qualified.”* (Male, 40 s, White British, CKD stage 1, high adherence) *“I preferred to start it straight away, just because after twelve weeks you might not want to do it anymore…or even again like your health might take a dip and you can't do those sorts of things, I think it’s quite a long time to wait.”* (Female, 20 s, White British, haemodialysis, moderate adherence)
	Remote trial assessment: Patient-reported outcome measures *“I struggled answering some of them because of where I am at in my kidney journey. Some of them I thought would be more relevant to me if I was doing dialysis or at a different stage.”* (Male, 40 s, White British, CKD stage 1, high adherence) *“It [the KDQoL-SF1.3] was not tailored to people who haven't had kidneys removed or haven't had dialysis, so there were one or two questions which I had to leave blank because it was to do specifically with things that didn’t apply to me.”* (Female, 60 s, mixed background, CKD stage 3a, very high adherence)
	Remote trial assessment: physical function assessment *“I did one [remote trial assessment] last week, and there was no difference with me doing it here or doing it [at the hospital], the only thing was because I was only on a phone, I wasn’t on a video call, and I wondered whether [the outcome assessor] thought that I was cheating.”* (Female, 50 s, Black British, CKD stage 5, high adherence) *“I think, I prefer it [remote assessment] over a video call, mainly because you get to see the person but then you also get to, like, do the assessment over the camera where someone can watch you and make sure like you're doing it correctly.”* (Female, teens, Asian British, peritoneal dialysis, moderate adherence)
Dissemination	*“I would like to see the results because it’s always interesting to see what is available. But then it comes back to the whole how friendly would it be made to me? Is it all going to be bar charts and numbers and statistics ?”* (Male, 30 s, Asian British, kidney transplant, moderate adherence) *“I’d be really interested to see what you learned from it and how people benefited or what the issues were. I think either a video or a presentation or something like that which we could join live or we could watch back.”* (Female, 50 s, White British, haemodialysis, low adherence)

#### Experiences of remote recruitment and assessment

Most participants found the online consent process straightforward but felt that telephone and face-to-face follow-up discussions remained important for informed consent. Emphasising the uniqueness and potential benefits of Kidney BEAM via diverse patient stories were suggested methods of increasing recruitment. Several people worried that allocation to the wait list control group would indicate they were not suitable for a physical activity-based intervention.

Remote trial assessments were universally described as simple and quick, and most people had no concerns regarding safety. The main challenges were lack of space and concerns regarding validity. The remote PROMS were less acceptable and described as repetitive, with some of the questions within the KDQoL-SF1.3 perceived as irrelevant.

#### Preferred methods for dissemination of the results

The majority of participants wanted to be informed about the results of the trial, and how these might impact clinical care. Information should be disseminated via email or video in an accessible manner.

### Perceptions of Kidney BEAM

#### Kidney BEAM within the CKD journey

Kidney BEAM was considered particularly relevant at the start of participants CKD journey. It was viewed as a trusted resource to address knowledge gaps and provide peer support. It was also felt to be useful following kidney transplantation, when participants were open to making lifestyle changes, but where there was a lack of specific guidance around effective and safe physical activity.

#### Inclusive

Kidney BEAM was described as positively framed and praised for offering standing and seated physical activity options, which allowed people to adapt the sessions to their ability. The video format was helpful for those participants managing fatigue, as it was easier to concentrate and the ability to pause the on-demand videos enabled periods of rest. The opportunity to choose from a wide range of physical activity types was also valued. Participants suggested providing additional CKD and life stage specific content, and more detailed information for people with a good existing understanding (Table 9).

**Table 9:** Illustrative quotations relating to participants’ perceptions of Kidney BEAM.

Kidney BEAM within the CKD journey	At the start of the kidney journey *“*I think for people who are new to kidney disease or, you know, maybe just had a transplant or whatever, maybe it would be helpful for them to get support and, you know, answer questions” (Female, 60 s, mixed background, CKD stage 3a, very high adherence) *“When the doctor is telling you all this stuff, you blank out. When I was first going there, I would always take someone with me who would listen and then after we used to sort of compare what bits I heard, what bits they heard, and try and build up a picture. So yes, having that sort of information on [Kidney BEAM] where you can link into it and…you’re not sitting there worrying.”* (Male, 50 s, White British, kidney transplant, moderate adherence)
	Post kidney transplantation ***“****After a few weeks of doing this, all I could think was if I had had this about when I first came out of that hospital and they said to me ‘right, you’ve got to do some exercise’, yeah, I’d have jumped at the chance. you’re sort of ‘what am I going to do that’s going to look after the kidney but isn’t going to push too hard?”* (Female, 60 s, White British, kidney transplant, very high adherence) *“I had my transplant a fortnight ago, on day two I asked to see the physio and I explained that I’d done this BEAM project and said ‘look, I know I can do some exercises, I want to be out of this bed’. I showed him some of the exercises I’d been doing and I got him to give me a few more to do. I got up and about a lot quicker than the other people who were transplanted at the same time. So, I was up and about and more to the point I was out of that hospital bed and out of that hospital in five days, which I don't think would have happened had I not been willing to be mobile and use some of those exercises…I think it paid off.”* (Female, 50 s, White British, haemodialysis, low adherence)
Inclusivity	Inclusive of a range of abilities ***“****What I liked about is that because I suffer from osteoarthritis in my knees that would mean some of the exercises might have been a bit challenging because of the pain that I suffer. There was [an instructor] who was doing sitting-down exercises. So sometimes I could intermix. If my knees had felt particularly good one day I’d do the complete course standing up, but on another day if they were hurting me then I would sit down. So, I thought that was excellent, I must admit. It really helped me because of my personal situation.” (Male, 50 s, White British, CKD stage 4, moderate adherence)* “They said to me do at least one of these programmes a week, but I probably could have done with some heavier weights.” (Male, 40 s, White British, CKD stage 1, high adherence)
	Inclusive of a variety of exercises *“I started to spot different [physical activity options] and I thought OK, I’ll have a go. I did the strength one…I did some yoga…and then I did some other stuff that I really enjoyed. I really liked trying all these different things out and experiencing things.”* (Female, 60 s, White British, kidney transplant, very high adherence) *“I was getting carried away in doing things like the yoga because I was like ‘oh this is really nice, I like this’… I was really happy with how it was laid out and what there was to offer.”* (Female, 30 s, White British, CKD stage 2, low adherence)
	Inclusive of people with fatigue *“I liked [the educational videos] because you just have to listen, and for me, it’s easier to listen than to read because I don't actually have to concentrate…it’s harder to concentrate sometimes, especially with kidney disease, you get tired really quickly.”* (Female, teens, Asian British, peritoneal dialysis, moderate adherence) *“With the on-demand ones, if it got too much, I could just pause it and then have a breather or sit down for a sec and then carry on when I could.”* (Female, 20 s, White British, haemodialysis, moderate adherence)
Areas for refinement to further enhance inclusivity	For people with different levels of knowledge ***“****I’ve been in the system for quite a long time, I do absorb a lot of information on it, but I was looking at [the educational videos] and thinking it’s very short on what it explains, doesn’t go into detail. Yes, people may not want to go into detail so maybe if you want more information at the end of it, maybe a link into something that would give more details.”* (Male, 50 s, White British, kidney transplant, moderate adherence) ***“****I would say I'm quite well versed in matters relating to kidney disease at this point, so I don't know what [Kidney BEAM] could tell me that I kind of don't already know.”* (Female, 20 s, White British, haemodialysis, moderate adherence)
	For people at different life stages *“Maybe if there was a blog post for students. Whenever I try and look for a kidney group or a blog or something, it’s always for older patients, patients who are already in jobs and middle-aged patients who look after families, and have their own job and careers. And they don't have any other educational commitments and they're not around my age.”* (Female, teens, Asian British, peritoneal dialysis, moderate adherence)

### Enhancing the opportunity to be physically active, and the challenge of time

Participants were managing hectic and unpredictable work and caring responsibilities. Those receiving dialysis were additionally managing high levels of treatment and symptom burden. Fatigue was exacerbated by these busy lifestyles and, together with fluctuations in health and ability, this made scheduling physical activity challenging. Kidney BEAM allowed participants to save time by reducing travel and offering flexible opportunities to be physically active. Despite this, they continued to report having limited time to engage with the DHI. Most participants focused on the physical activity sessions and personally relevant educational topics and were unaware of the breadth of content available.

Suggestions for refinement centred around helping participants to maximise their available time. Kidney BEAM could be made easier to navigate, and participants suggested that a short video ‘tour’ and increased telephone or online support would also help. They also felt it was important to highlight new content and provide guidance on key content. Shorter session durations, a more extensive live class timetable and greater leeway with live class bookings were also suggested (Table 10).

**Table 10:** Illustrative quotations relating to enhancing the opportunity to be physically active, and the challenge of time.

Barriers to physically active	Hectic lifestyles *“My biggest challenge which will probably be borne out later is…finding the time for all this. I get on and do what I need to do and then I didn’t spend too much time exploring everything else”* (Male, 40 s, White British, CKD stage 1, high adherence) *“My work became so heavy; I was running three different shops and running back and forth. Work is only just starting to calm down at the moment, so is that sort of oh at some point I’ll get back on and do a couple more [classes].”* (Male, 50 s, White British, kidney transplant, moderate adherence)
	Treatment and symptom burden *“I work and then I have dialysis in the evenings, I then only have about three or four days in which I'm free in the evenings and a lot of them times I'm just so tired that it’s really hard to find the energy to exercise. I struggled with finding the energy in the right amount of time to do it.”* (Female, 20 s, White British, haemodialysis, moderate adherence) *“I really didn’t have time, it was like trying to find time in the week that coincided with various things I volunteer for and I'm a carer and, you know, hospital appointments”* (Female, 60 s, mixed background, CKD stage 3a, very high adherence)
Enhanced opportunities to be physically active via Kidney BEAM	Accessibility and flexibility of Kidney BEAM *“I didn’t have to pack my stuff up and go trotting off to a gym somewhere and hospital to find a parking space or get on a bus, it was here in my own home, it was comfortable…it was convenient.”* (Female, 60 s, mixed background, CKD stage 3a, very high adherence) *“I found them quite accessible, so you didn’t need any particular kit to do them, you didn’t need to have any specific skills particularly.”* (Male, 40 s, White British, stage 3a, low adherence) *“A lot of [my exercise] I was doing at awkward hours. At one point I done one at three o’clock in the morning, so [on demand classes] made it easier for me.”* (Male, 50 s, White British, kidney transplant, moderate adherence) *“I found them quite good in that it was understandable language, it went into enough depth to sort of make you understand what was happening and the issues, but it wasn’t so complicated to get confusing at any point, so I did find those quite useful. You could watch them in between things, you can sort of just click and you're not dedicating too much time.”* (Male, 40 s, White British, stage 3a, low adherence)
Ongoing challenges to using Kidney BEAM due to lack of time	Limited time to engage *“I lead a very, very busy lifestyle, you know, and I followed the course for the patients who had had a kidney transplant, specifically. I did not look at any other, anything, so I just looked at that one because that seemed to be the one that fits me.” (Male, 50 s, White British, CKD stage 4, moderate adherence)* *“I wasn't aware of [the blogs and groups] so maybe that could do with making a bit more obvious.”* (Male, 40 s, White British, CKD stage 1, high adherence) *“I wish I knew [about additional content on Kidney BEAM]. I genuinely wish I knew, because I’ve been thinking about doing yoga for a while, and, with the high-intensity training, it’s something that I’ve been thinking about because people say it’s really, really good for weight loss.”* (Male, 30 s, Asian British, kidney transplant, moderate adherence)
Areas for refinement to further to optimise the time available	Simpler navigation *“The overall kind of layout was good, if not a little bit confusing to navigate!”* (Female, 20 s, White British, haemodialysis, moderate adherence) *“I had a little bit of difficulty negotiating the amount of stuff on there, so for me I think they were directing me to one particular exercise programme and there’s a lot of other exercise programmes on there and I think the first one I did I think I probably did the wrong one, I loved it, but I did a boxing one!”* (Female, 50 s, White British, haemodialysis, low adherence)
	Support with navigation *“Maybe do like a video, a multimedia thing where you can see this is what it looks like, a quick tour of the platform maybe.”* (Male, 40 s, White British, stage 3a, low adherence) *“I think for me personally, to have somebody talk me through it while I did it, so if I say, had my tablet and I had someone in my phone talking to me would have been perfect.”* (Female, 50 s, Black British, CKD stage 5, high adherence)
	Highlighting content *“The front page, that could be a place where you could update or there could be when you log in, an update on classes that are online or new blog posts or something like that, just an announcement thing.”* (Male, 40 s, White British, CKD stage 1, high adherence) *“An introductory how to use the platform, and maybe that needs to contain something about you can chat with the instructors here or you can interact with groups there because I'm not sure I picked all that up.”* (Female, 50 s, White British, haemodialysis, low adherence)
	Guidance on what to select *“My first thing was “Well where do you want me to go? What ones do you want me to do?” There is a big option, but a guide to “maybe try these ones first” would have been helpful at that time”.* (Male, 50 s, White British, kidney transplant, moderate adherence) *“I think for the process of the study, they probably need to be really, really clear. I went into an exercise programme and thought that I was on the right one. If they want somebody to follow a specific pattern of exercise, they need to be really sure that they're on the right one, I mean, for me, it didn’t make any difference, but I'm guessing they could potentially do some harm to themselves depending on what they'd had done. You do tend to go a bit wandering once you get on and you think ooh that’s interesting!”* (Female, 50 s, White British, haemodialysis, low adherence) *“Looking for like the classes in Kidney Beam…was kind of confusing because I didn’t know which one to go into”* (Male, 50 s, White British, Peritoneal dialysis, moderate adherence)
	Shorter physical activity sessions *“There were a couple of times when I [thought] “Oh I only really want to do twenty minutes” so I didn’t do all of the session in one go, but you’ve made it in that way that you could do that. But yes, it’s sort of jumped from short, up to suddenly forty minutes on some of them, so maybe sort of a bit more variation in the length, so if you have only got half hour, you can do the half hour. If you’ve got that extra bit of time, you can do the longer one.”* (Male, 50 s, White British, kidney transplant, moderate adherence)
	Live classes: extended timetable *“Having the live classes may be done of an evening might encourage more people either to link into classes a bit easier.”* (Male, 50 s, White British, kidney transplant, moderate adherence) *“If sometimes they can do two classes in a day maybe, so like one at eleven and maybe one at four or five, because the working day finishes at four/five o'clock, those who do have work can do it after, can come after.”* (Female, teens, Asian British, peritoneal dialysis, moderate adherence)
	Live classes: booking and reminders *“A little bit of leeway because often I wouldn’t know if I could commit to a class. I probably should have booked it in earlier but then sometimes I’d just miss out because it was 20 s after 11 o’clock and I tried to book and the administrations closed. If there was like a minute or two leeway that might help.”* (Male, 40 s, White British, CKD stage 1, high adherence)

### Enhancing motivation to be physically active

Despite the challenges with attending the live classes due to lack of time, those able to attend found them highly engaging. The instructors were perceived as warm, inclusive and knowledgeable. Areas for enhancement included making the relevance of increased physical activity on CKD more evident and offering one-to-one reviews. Participants with higher adherence underlined the importance of the community and accountability created by the live classes, which fostered ongoing participation. Text message reminders, mandating more than two classes per week, and using personal goals were highlighted as methods for further enhancing accountability. People with lower adherence were more likely to describe feeling self-conscious.

The physical activity diary was described as a motivational means of tracking progress and increasing ‘offline’ physical activity. Participants felt the ability to sync the diary with wearable monitors would reduce burden. Introducing summary analytics to help them understand how their physical activity levels are influenced by factors such as symptoms and time of the day was also suggested to support activity planning.

#### External support for Kidney BEAM

Other sources of support and motivation were family, friends and the Kidney BEAM team. Many participants completed the sessions alongside a family or friends and had subsequently been encouraged by them to engage in offline physical activity.‘Check-ins’ by the Kidney BEAM team were also described as supportive and useful for increasing accountability (Table 11).

**Table 11:** Illustrative quotations relating to motivation to be physically active.

Influences of live kidney BEAM classes on motivation	Live classes: instructors *“The instructors were pretty sympathetic and if there were things you couldn't do, you know, you'd go and sit down, nobody sort of pointed their finger and said ‘Oh what are you doing, why are you sitting down, stand up, do it’, you know. Because I don't personally like bossy instructors, you know, that sort of shout ‘come on, you can do it, get up, get up, move those arms, move those arms!”* (Female, 60 s, mixed background, CKD stage 3a, very high adherence) *“They were engaging, it wasn’t boring, and it wasn't regimented. She made you feel comfortable, so that was good.”* (Female, 50 s, Black British, CKD stage 5, high adherence) *“They were never going to be drill sergeants, but it was nice to have that kind of warmth about them and it was also just always saying “Try and push. Try and get to the last one” You know. It’s [also] really nice to feel included…one particular trainer said, “Drink less, if you’re on dialysis” or “be careful if you have a fistula” it’s just that idea of feeling included.”* (Male, 30 s, Asian British, kidney transplant, moderate adherence)
	Live classes: community *“Having other people makes it feel more of a group activity, that you’re not just doing it by yourself, which I think especially during lockdown and things like that when we’re not seeing as many people is good and it makes you feel a bit more supported in doing it.”* (Male, 40 s, White British, CKD stage 1, high adherence) *“I think sometimes that’s the biggest benefit of some exercise classes is not the exercise; it’s giving people human contact who maybe don't have that opportunity in real life.”* (Female, 60 s, mixed background, CKD stage 3a, very high adherence) *“Listening to the discussions and hearing that people are having the same problem, there’s a lady there practically my mirror image, and so that’s kind of comforting in a strange way.”* (Female, 50 s, Black British, CKD stage 5, high adherence)
	Live classes accountability *“In some ways, if you're doing a live class you're almost forced to show up. With an on-demand, you think oh I’ll do it a bit later, whereas if it’s a scheduled class you are sort of forced into doing that.”* (Male, 40 s, White British, stage 3a, low adherence) *“Being able to see the instructor demonstrate how to do it with the live classes, you feel a bit more accountable to actually do it. I would prefer to do them live because I think just seeing the other people doing it as well, it feels like – again for accountability reasons like you’ve got to show up and put in the effort.”* (Male, 40 s, White British, CKD stage 1, high adherence) *“*I'm not particularly interested in spending lots of time speaking to people who are ill… I find that some of the interactions I have…not similar, I've met very few people who have worked all the way through haemo and dialysed at home while they're working. I just haven't come across them and some of the people I have met have been a lot iller than I was– I didn’t emotionally and mentally feel ill” (Female, 50 s, White British, haemodialysis, low adherence)
Areas for refinement to further enhance motivation in the live classes	For CKD stage *“I'm curious as to why exercising would help a kidney. I can understand exercising helping keep your joints supple, keep your heart…pumping away, but the kidneys are tucked away, it’s just helping fluids and stuff, how can exercise help a kidney?”* (Female, 60 s, mixed background, CKD stage 3a, very high adherence) *“I just thought it would be something that would help me with my [peritoneal dialysis] catheter and my stomach and what things I can do. That’s all that bothers me to be honest, what I can lift and how I can lift it. I just want to be normal. I just didn’t understand the link between an exercise class and kidney failure.”* (Male, 50 s, White British, Peritoneal dialysis, moderate adherence) *“I think as it’s aimed at kidney patients…I would have thought, depending on what type of dialysis you were on and where you were in your treatment, there might be some very specifics around, you know, you may not want to do too many stomach exercises and sit down and stand-ups if you’ve got a line in your tummy.”* (Female, 50 s, White British, haemodialysis, low adherence)
	Individual reviews *“I don't know whether you could do some Q&A things where if you have specific questions about particular things or particular modifications you might need with some of the exercises…it might be good to have an avenue to say ‘I can't do this, what would you suggest I do as an alternative?’”* (Male, 40 s, White British, stage 3a, low adherence) *“It would have been nice… to give each member of the class…a little fifteen-minute slot on their own just so you can look specifically at them, how they do the exercise…just check if everybody’s doing it OK as best you can.”* (Female, 60 s, mixed background, CKD stage 3a, very high adherence)
	*Minimum requirements and setting goals* *“I think if you’d mandated three sessions a week from the outset, I probably would have made it work.”* (Male, 40 s, White British, CKD stage 1, high adherence) *“I like a goal and I'm better with a target! I'm better with ‘do three a week’ than if you just said to me ‘have a look at it when you like.’ I may not have done it quite so often.”* (Female, 50 s, White British, haemodialysis, low adherence)
Influences of the physical activity diary on motivation	*“[The diary] was good because [it was] another form of motivation seeing what you’ve done and…being able to track. You know what you’ve done and what you haven’t done. I think also showing the aggregate of how much you did this week versus last week is a good feature.”* (Male, 40 s, White British, CKD stage 1, high adherence) *“[The diary] was useful because it’s quite nice to be able to look back through what you’ve done”* (Female, 20 s, White British, haemodialysis, moderate adherence)
Areas for refinement to further enhance the physical activity diary	*“Not that I’m overly analytical but it would be interesting to see OK, well at this is the point in the day – do I work out at eight or do I work out at ten? And then maybe I can kind of arrange things in a certain way.”* (Male, 30 s, Asian British, kidney transplant, moderate adherence) *“Quite often it’s all there on your phone, isn't it, you could have just copied it off and popped it in.”* (Female, 30 s, White British, CKD stage 2, low adherence) *“If you could add notes for yourself like for different days it’s quite nice to be able to look back and go ‘Oh well I struggled with that but now it’s easier’, you know, so it’s always good to see that sort of progress. When you’ve got symptoms of fatigue that’s limiting you, it’s quite useful to see how that might have changed or influenced things”* (Female, 20 s, White British, haemodialysis, moderate adherence)
External support to enhance motivation	Family and friend support *“Since Kidney Beam started [my friends have] encouraged me to go play badminton with them and they're…giving me a bit of help with exercise and tips and stuff and how to make it easier for me.”* (Female, teens, Asian British, peritoneal dialysis, moderate adherence) *“My partner watched a few of the informational videos and joined in with a few of the exercises if we got them up on the big telly.”* (Female, 20 s, White British, haemodialysis, moderate adherence)
	Kidney BEAM team support *“I felt like I knew [the Kidney BEAM administrator] – I’d never met her – never spoken to her – so she’s had email contact from time to time and say ‘obviously you’ve not been on the platform, is everything OK?’, and I said ‘well actually I've got Covid’ and it was really helpful just knowing that when I couldn’t do something or when there was a problem that she was at the end of an email and she had a sort of a chatty, friendly style, so you didn’t feel like you were being told off!”* (Female, 50 s, White British, haemodialysis, low adherence) *“A few people got in touch and they were saying “You haven’t been on. Is everything OK?” And I think that’s nice because it wasn’t kind of like a “What’s going on? You haven’t turned up to the gym, what’s going on?” like I’m assuming a personal trainer would, it’s more “Is everything OK?” Because we actually could have been on a visit to the hospital or something could have happened. And I think that tone was carried really well throughout.”* (Male, 30 s, Asian British, kidney transplant, moderate adherence)

### Integrated mixed methods analyses

Integrated analyses highlight that the ‘go’ criterion for progression to the definitive trial was met for outcome acceptability, alongside the ‘change’criteria for recruitment, intervention acceptability and retention. Quantitative and qualitative analyses were both complementary and confirmatory, indicating that progression to the definitive trial would be feasible and that Kidney BEAM is acceptable, following adaptations to increase recruitment, reduce loss to follow-up and promote increased engagement. The integrated analysis and the planned adaptations to the trial and intervention are outlined within Table 12, with the rationale for suggested intervention refinements included within in Supplementary Material 4.

**Table 12:** Joint display of quantitative and qualitative results, with corresponding adaptations made to the trial and Kidney BEAM intervention.

	Progression criteria status	Feasibility trial results	Qualitative results	Adaptations to the trial or to Kidney BEAM
Recruitment	Amber	• Pilot period sites were able to recruit a median of 6 (IQR 3–13) participants per month • The majority of the sample were White British (n = 29, 69%), and non-frail (n = 42, 100%)	• Most participants were motivated to take part by a pre-existing desire to be more physically active. Altruism was also a key motivator to participate but seemed to result in lower levels of adherence • Online consent needs to be supported with telephone and face to face support, including careful discussion of the randomisation process	• Further research into reach amongst those who declined, particularly those with protected characteristics, to be conducted as part of a planned sub study • Add further centres to increase monthly recruitment rates • Follow-up non-responders • Provide a script to support the recruitment of people not already contemplating becoming more active (see Supplementary Material 5)
Outcome acceptability	Go	N = 42 (100%) completed the KDQoL SF1.3 at baseline and n = 35 (83%) at 12 weeks	Remote trial assessments were seen as quick and easy, but careful explanation regarding the PROMs collected was needed to increase understanding and to support accurate completion	No adaptations required
Intervention acceptability	Amber	N = 13 (59%) completed at least 1 session of physical activity per week over the pilot period	• Whilst Kidney BEAM enabled people to address some of the challenges associated with lack of time and motivation, these barriers continued to be highlighted as barriers to engaging with BEAM • Reducing the burden associated with navigating the DHI, and uploading physical activity, providing additional support, reminders and tailoring were all identified as methods to increase engagement with the platform	• Provide more CKD stage and life stage specific content • Provide more detailed information for those who are not newly diagnosed • Enhance ease of navigation via support and ‘how to videos’ • Highlight new content on the site • Provide advice on the key aspects of Kidney BEAM to engage with
Loss to follow up	Amber	Overall dropout rate (pre-baseline and during the trial) was n = 15 (30%, 95% CI 18–45%). Dropouts at follow up were not connected to the acceptability of Kidney BEAM, but those pre-baseline may have been related to their understanding of the trial	Reasons for dropout were not explored with this group, all of whom completed the intervention	

## Discussion

The results of this pilot trial suggest that progression to a definitive trial is warranted, provided adaptations are made to enhance recruitment and retention rates. The progression criterion for outcome acceptability was achieved and there were no adverse events. Feedback regarding the acceptability of Kidney BEAM was positive, with suggested refinements focused on supporting people maximise their available time.

This trial is the first to demonstrate the feasibility and acceptability of a physical activity and emotional well-being DHI for people with CKD. The pre-specified progression criteria supported continuation and identified adaptations to enhance recruitment and retention. The recruitment rates observed in this trial are lower than those seen in other multi-centre trials of physical activity programmes delivered across the CKD spectrum, which range from ~ 41 to 69%^42–44^. The reason for this discrepancy is likely due to these trials using face to face recruitment, rather than remote, recruitment and the interventions being highly structured and supervised, rather than delivered entirely online. Importantly, these interventions have failed to have been widely implemented within routine practice because the workforce and expertise required to do this are not routinely available to people with CKD^45^. When compared with other trials of self-management and physical activity DHIs for long-term conditions, rates of refusal within this pilot are comparable (also ~ 32%)^46^. Optimising the recruitment for Kidney BEAM and ensuring the wide reach and acceptability of this intervention, using the learning gathered from this internal pilot and the subsequent definitive trial, will ensure that safe, scaleable and effective physical activity and self-management intervention may be delivered to a demographically and geographically diverse population, bridging this significant translational gap.

Notably, the trial was also conducted during the pandemic, when many centres were not actively recruiting to trials. This undoubtedly contributed to the lower-than-expected recruitment rates. Trials planned during this time required protocols that were safe and deliverable, whilst also maintaining scientific validity and integrity^47,48^. A shift to remote trial delivery increased exponentially during this period, paving the way for more remote trials in the future. The results of our trial demonstrated challenges with converting potential participants into enrolees, supporting the findings of a recent systematic review, where conversion rates were higher in those recruited ‘offline’, and in some cases led to the recruitment of ‘atypical’ populations^49^. Existing evidence recommends a combination of on, and offline recruitment be used to derive a representative sample^49^ and a tool kit for remote trial delivery has been published since the inception of the Kidney BEAM trial, recommending staff training, follow-up and support for participants being recruited online^47^. The learning from the pilot has led to several adaptations to the recruitment process which are outlined in Table 12. The adaptations needed to achieve the required sample size for the definitive trial included: the addition of further centres, following-up non-responders, and a script to support the recruitment of people not already contemplating becoming more active (Supplementary Material 5). Similar recruitment strategies are effective in trials of DHIs in other long-term conditions^10^ and may also address the high rates of non-responders and post-randomisation withdrawals.

Similarly, retention rates did not reach our a priori criteria. We were not able to explore this further within the interviews, which were conducted with participants who completed the trial. Other studies have reported similar challenges, and rates of retention of around 48%, within online trials and DHIs^50,51^. Considering this, our retention rate of 70% compares favourably, particularly for a physical activity intervention which requires significant engagement and motivation. Loss to follow up in online trials and digital interventions appears to be positively influenced by a range of factors including referral from a clinical team member, targeting motivated participants, incentivisation, nudges and reducing study complexity^50,51^. These strategies may be employed to increase retention, but must be balanced with the potential dangers of selecting participants which are not representative of all people with CKD.

The baseline characteristics of those recruited were largely representative of the UK CKD population^52^, providing reassurance relating to the potential external validity of the definitive trial. Whilst the demographics of those who declined were similar to those who participated, frailer participants were not recruited. The incidence of frailty is approximately 75% within the CKD population^53^ and physical activity is an important component of frailty management^54^. Physical activity DHIs for frail older people are effective^55^ and Kidney BEAM may represent an important intervention for this particularly vulnerable group, but it remains uncertain whether the challenges to recruiting this group are reflective of the acceptability of Kidney BEAM, or because the research teams inadvertently acted as gatekeepers to the trial, as has been observed in other trials^10^.

Kidney BEAM was well received due to its inclusivity, accessibility and flexibility. Similar findings have been reported in DHIs for other long-term conditions, where choice over how and where support is accessed increased a sense of control and reduced anxiety^8,10^. Kidney BEAM also increased participants’ opportunity and motivation to be physically active, however, lack of time continued to be the predominant ongoing barrier to engagement. Competing priorities has been highlighted as a major barrier to engagement with DHIs in other long-term conditions^10^. Although the ‘go’ criteria for intervention acceptability was not achieved, adherence to kidney BEAM (58%) compared favourably with physical activity DHIs for other long-term conditions (55%)^56^ and face-to-face renal rehabilitation programmes (59%)^57^.

Suggested refinements focused on further enhancing participants’ opportunity and motivation to engage, via individual tailoring. Allowing users to create personalised goals, customise the level of the information within the DHI and receive personalised reminders, have all been shown to increase engagement and effectiveness in other long-term conditions^8,56^ and DHIs for people with CKD^9^. DHIs lend themselves well to tailoring^10^, and the feedback received from participants will be used to further personalise the content on Kidney BEAM in the future. The physical activity diary and goal-setting features were valued for helping participants track personal progress. Enhancing this by syncing the diary with wearable activity trackers and providing personal analytics has been shown to increase adherence across a range of other long-term conditions by supporting users to gain new understanding about how to manage their condition, whilst also reducing burden^10,56^.

Participants highlighted that ease of use was key to increasing meaningful engagement. Intuitive and easy-to-use DHIs are important for securing initial engagement^8,10^ and sustained use over time^56^. In a similar manner to recruitment to the trial, healthcare professional support was also important for the delivery of the DHI, with engagement highlighted as important sources of motivation. The integration of healthcare professional support with DHIs has been consistently associated with increased adherence in other long-term conditions^56^, via increased self-confidence, development of skills and reduced isolation^56,58^.

The community created via Kidney BEAM was an important motivator for some participants, but less appealing to others. This ambivalence has been observed in other trials and is related to individual preferences^8,56^ Kidney BEAM allowed participants to select an option which suited their personal preferences, enhancing acceptability and reducing the potential for negative social comparison^56^. Interestingly, family and friend support was also crucial for many participants, which was not anticipated in the development phase. Previous research suggests that targeting these wider support networks is important to increasing uptake^10^ and the results of this current trial indicate that it can also promote ‘offline’ activity outside of the DHI.

### Strength and limitations

To our knowledge, this trial is the first to examine the feasibility and acceptability of a remote trial of a CKD-specific DHI focused on physical activity and emotional well-being. A key strength is a strong emphasis on the co-production of the intervention using established, theory-informed approaches which have previously been lacking within this field^9^. An acknowledged limitation is the lack of exploration of the views of researchers and withdrawing participants which would have provided further useful insight.

One of the reasons the ‘go’ criteria may also not have been achieved was that not all people may have been able to access and use an English language DHI. Existing reviews indicate that although DHIs may lead to improvement in clinical outcomes across a range of long-term conditions, they are most often utilised by digitally and health literate people with access to technology^10,59^. Up to 20% of the general population do not possess fundamental digital skills, resources, motivation and confidence needed to effectively use DHIs^8,10,60^. Digital exclusion can perpetuate health inequality^60^ and, given that the COVID-19 pandemic has accelerated the introduction of DHIs with routine care^59^, exploration of digital exclusion in relation to online physical activity and emotional wellbeing self-management programmes is warranted. Not all patient populations are affected in the same way, or experience the same barriers, making understanding the perspectives of those who do not participate in the trial particularly important^60,61^. Strategies that have proven beneficial to engaging frailer participants in other evaluations of DHIs include providing access to technology, providing support to increase ‘readiness’ to use the intervention, addressing concerns about injury, social isolation and security and avoiding stigmatisation^62,63^. As part of the evaluation of Kidney BEAM, we are conducting a qualitative sub-study^12^ which will explore these challenges in people who declined to participate in the trial. The findings of this sub-study, alongside a substudy specifically examining the role and effectiveness of Kidney BEAM during the intradialytic period for people receiving HD, will more comprehensively inform strategies to address inequality of access and to improve the reach of Kidney BEAM.

Additionally, some of the qualitative researchers were known to the participants from their roles as physiotherapists delivering the classes or as the Principal Investigator at one of the sites. Although this may have influenced their responses, this risk was mitigated by ensuring participants were interviewed by a researcher unknown to them. In some instances, these dual roles were helpful, as knowledge of the trial and DHI allowed researchers to provide further prompts and explanations.

## Conclusion

A definitive trial to evaluate the clinical and cost-effectiveness of Kidney BEAM feasible, with adaptation to increase acceptability. The results of this definitive trial will provide robust evidence for the role of DHIs in supporting physical activity and self-management in people with CKD. This will address an area of unwarranted variation in care and potentially lead to a step change in the clinical management of this population.

### Supplementary Information

Supplementary Information 1.

Supplementary Information 2.

Supplementary Information 3.

Supplementary Information 4.

Supplementary Information 5.

## Data Availability

The data underlying this article will be shared on reasonable request to the corresponding author.

## Abbreviations

AE: Adverse events
APEASE: Affordability, practicality, effectiveness, acceptability, safety, equity
CKD: Chronic kidney disease
DHI: Digital health intervention
HRQoL: Health-related quality of life
MoSCoW: Must have, should have, could have and would like
NHS: National Health Service
PPI: Patient and public involvement
PROMS: Patient reported outcome measures
RCT: Randomised controlled trial
SAE: Serious adverse events
STS60: Sit-to-stand in sixty seconds
