# Supplementary material for: The development and internal pilot trial of a digital physical activity and emotional well-being intervention (Kidney BEAM) for people with chronic kidney disease
Source: Sci Rep. 2024 Jan 6;14:700. doi: 10.1038/s41598-023-50507-4 (PMC10771473; doi:10.1038/s41598-023-50507-4)
Supplement: Supplementary file 1 — Supplementary Information 1. [file 41598_2023_50507_MOESM1_ESM.docx]

### Detailed overview of the development of the Kidney BEAM intervention

#### Initial development phase

The initial development of Kidney BEAM included four iterative stages, which occurred rapidly over an intensive four-week period. Development was informed by the key principles outlined in the INDEX and DHI guidance.^1 2^ A diverse range of stakeholders were closely involved in co-producing the intervention throughout. These included fifteen expert researchers and clinicians with backgrounds in rehabilitation, physical activity, nephrology and digital health, five web developers, six partners from kidney charities (see Supplementary material 1 for details) and six stakeholders with expertise through lived experience of CKD (*n*=3, 50% male; 53±17 years; *n*= 3,50% White British; *n*=2; 33% pre-dialysis CKD, *n*=2, 33% dialysis, *n*= 2, 33% transplanted). Whilst the shielding and lockdown restrictions imposed by the pandemic at the time precluded face-to-face interaction, the group met regularly using a series of meetings supplemented with the use of an online messaging app (Slack Technologies, LLC). The principles of co-production and participatory design for DHI were followed throughout.^3^ ^4^ All perspectives were of equal importance and stakeholders shared the ability to make key decisions with the research team, with final recourse to the Chief Investigator.

1. Understanding the need

Whilst the restricted timescale for development prohibited any primary research during the development phase, existing and ongoing research relating to DHIs for physical activity was reviewed. Following this, barriers and facilitators to physical activity were reviewed and matched to the COM-B model within a behavioural analysis.^5-10^ The COM-B model posits that three components need to be present for a behaviour to occur. These are: capability (the psychological and physical capacity to engage in the behaviour), opportunity (the physical and social factors that are conducive to the behaviour), and motivation (internal processes which influence decision-making and behaviours, including both reflective and automatic processes).^10^ ^11^

Several common barriers and motivators across all CKD stages and kidney replacement therapy modalities were identified from qualitative and survey data and these are summarised in Table 2. Reflective motivation and physical opportunity occurred most frequently, implying that facilitating the opportunity to become physically active and leveraging behaviour change techniques to enhance planning and evaluation could promote physical activity.

Table 2. Behavioural analysis: known barriers and facilitators to physical activity across CKD stages and kidney replacement therapy modalities mapped to the COM-B model.

| **Barriers and facilitators to physical activity in people with CKD** | **Capability** | | **Opportunity** | | **Motivation** | |
| --- | --- | --- | --- | --- | --- | --- |
|  | Physical | Psychological | Physical | Social | Reflective | Automatic |
| Reduced mobility and function (perceived and actual) ^abe^ |  |  |  |  |  |  |
| Symptoms arising from CKD and its treatment ^abe^ |  |  |  |  |  |  |
| Presence of comorbidities and changes in health ^ab^ |  |  |  |  |  |  |
| Lack of awareness of information about physical activity benefits and implementation ^abcde^ |  |  |  |  |  |  |
| Lack of opportunity to be physically active due to the burden of treatment ^a^ |  |  |  |  |  |  |
| Lack of opportunity to exercise due to catheter and vascular access ^ae^ |  |  |  |  |  |  |
| Lack of accessible or suitable environments in which to exercise ^abc^ |  |  |  |  |  |  |
| Lack of exercise equipment ^a^ |  |  |  |  |  |  |
| Emotions (sadness, helplessness, anxiety) ^a^ |  |  |  |  |  |  |
| Fears relating to safety (blood pressure stability, damage to vascular access, injury, exacerbation of condition/ symptoms, falls) ^abce^ |  |  |  |  |  |  |
| Peer and family support for physical activity ^abcd^ |  |  |  |  |  |  |
| Healthcare professional support for activity and prioritisation within CKD care ^abc^ |  |  |  |  |  |  |
| Desire to improve physical condition, independence and maintain normality ^abce^ |  |  |  |  |  |  |
| Observing improvements and achieving goals ^bcd^ |  |  |  |  |  |  |
| Feeling guilty for not exercising ^bc^ |  |  |  |  |  |  |
| Enjoyment of physical activity and choice ^bc^ |  |  |  |  |  |  |

^a^ barriers and facilitators to physical activity identified by people with end-stage kidney disease^6^; ^b^ barriers and facilitators to physical activity identified by people with pre-dialysis CKD^7^; ^cd^ barriers and facilitators to physical activity identified by kidney transplant recipients^8 9^; ^e^ barriers and facilitators to physical activity identified by people receiving peritoneal dialysis^5^.

2. Identification of intervention functions

Potential levers for change identified by the behavioural analysis were linked with intervention functions likely to be effective, using the Behaviour Change Wheel (BCW).^11^ The BCW is a systematic approach which has been widely used to develop physical activity and rehabilitation interventions for people with long-term conditions.^11^ The wheel links COM-B components to nine intervention functions, revealing appropriate strategies for supporting and maintaining behaviour change.^11^ The following candidate intervention functions were identified: (i) training: imparting practical skills; (ii) enablement: increasing means / reducing barriers to increase capability or opportunity (iii) persuasion: using communication to induce positive or negative feelings or stimulate action; (iv) education: increasing knowledge or understanding; (v) incentivisation: creating an expectation of reward; (vi) environmental restructuring: changing the physical or social context; and (vii) modelling: providing an example for people to aspire to or imitate (Table 3).

These functions were then considered in relation to their affordability, practicality, effectiveness and cost-effectiveness, acceptability, safety and equity (potential for impact upon health inequalities), collectively known as the APEASE criteria.^10^ Consideration of contextual fit was also key for Kidney BEAM to be readily implemented within the current NHS clinical landscape.^1^ Extensive engagement with NHS England, NHS Digital, Kidney Care UK, Kidney Research UK, The National Kidney Federation, the UK Kidney Association, the Chartered Society of Physiotherapy and the UK Kidney Research Consortium MedTech Group was undertaken to understand the current context into which Kidney BEAM would be implemented. This engagement highlighted the need for the online platform to meet the key Digital Technology Assessment Criteria (DTAC), including clinical safety data protection, technical security, interoperability and usability and accessibility standards. Compliance with these standards allowed for the consideration, and publication, of the Kidney Beam case study within the NHS Digital Renal Playbook.^12^ During the pilot, Renal Getting it Right First Time^13^ was published and The Renal Services Transformation Programme^14^ initiated, which highlighted the need for holistic psychosocial and physical rehabilitation services, and this provided further opportunity to ‘scale up’ digital health interventions such as Kidney BEAM for greater national impact and future sustainability.

Table 3. Intervention strategies and intervention functions linked to COM-B components to promote increased physical activity in Kidney BEAM

| **Intervention strategy used within Kidney BEAM** | **Behaviour Change Technique** | **Intervention functions** | **COM-B** |
| --- | --- | --- | --- |
| Offer a range of physical activity classes and videos for people of different abilities and with different co-morbidities | - Demonstration and instruction of the behaviour via live sessions and videos - Encouragement of practice via repeated interaction and reminders - Monitoring and feedback on the behaviour - Self-monitoring of the behaviour using the activity diary | - Training | Physical capability |
| Provide accessible information about physical activity benefits, how to start and maintain a physical activity programme, specific to different stages of CKD and forms of kidney replacement therapy | - Information about the benefits of physical activity and the effects of inactivity specific to CKD and related co-morbidities - Information/ specific physical activity advice according to kidney replacement therapy modality | - Education | Psychological capability |
| Offer physical activity options that can be done at home with minimal space and equipment | - Restructuring the environment to remove the need for equipment - Identifying items around the home that could be used for resistance training | - Enablement | Physical opportunity |
| Increase access to peer and healthcare professional support for physical activity | - Opportunities to see peers undertaking physical activity during live classes - Social support during class from peers and credible healthcare professionals, and in the education, sessions following a live class - Peer support groups | - Modelling - Enablement | Social opportunity |
| Address beliefs about the influence of physical activity on symptoms and comorbidity | - Information on common symptoms in CKD (e.g., fatigue) and how physical activity might influence this delivered by credible sources (HCPs and people with CKD) - Information on common comorbid conditions and the influence of physical activity - Problem-solving and action planning for physical activity barriers - Self-monitoring via the physical activity diary - Sharing of experiences around symptoms and comorbidity between peers during live classes | - Education - Enablement - Persuasion - Modelling | Reflective motivation |
| Address beliefs about the ability to be physically active with mobility and functional restrictions | - Information on current physical activity guidelines - Information on the benefits of physical activity specifically for those with low levels of functional ability, delivered by credible sources - Support and options via video and live classes for people with lower levels of ability or functional restrictions - Demonstration of behaviours and linked back to tangible functional benefits - Ability to observe peers during a live class | - Education - Enablement - Persuasion - Modelling | Reflective motivation |
| Provide feedback on improvements in physical condition | - Feedback via diary and the assessment process - Support participants to undertake SMART goal setting - Self-monitoring of progress with goals | - Education - Persuasion - Incentivisation | Reflective motivation |
| Provide support to deal with challenging emotions arising from living with CKD | - Peer support groups - Kidney psychotherapist/counsellor-led groups | - Enablement | Automatic motivation |
| Address concerns relating to the safety of physical activity | - Information regarding common fears relating to physical activity, delivered by credible sources - Feedback on technique and suitable progression within live classes - Demonstration of safe techniques via video and during live classes | - Education - Persuasion - Modelling | Automatic motivation |

1. Identifying content and implementation options

Identified intervention functions were then linked to appropriate behaviour change techniques (BCTS) using the behaviour change taxonomy.^15^Appropriate BCTS were selected by the stakeholder group based on the APEASE criteria,^10^ their existing evidence base and whether the selected BCT was appropriate for use within a DHI. Intervention functions and their linked BCTs are outlined in Table 3. A logic model outlining the proposed mechanisms by which Kidney BEAM influences intermediate outcomes and long-term goals (HRQoL) is outlined in Figure S1.


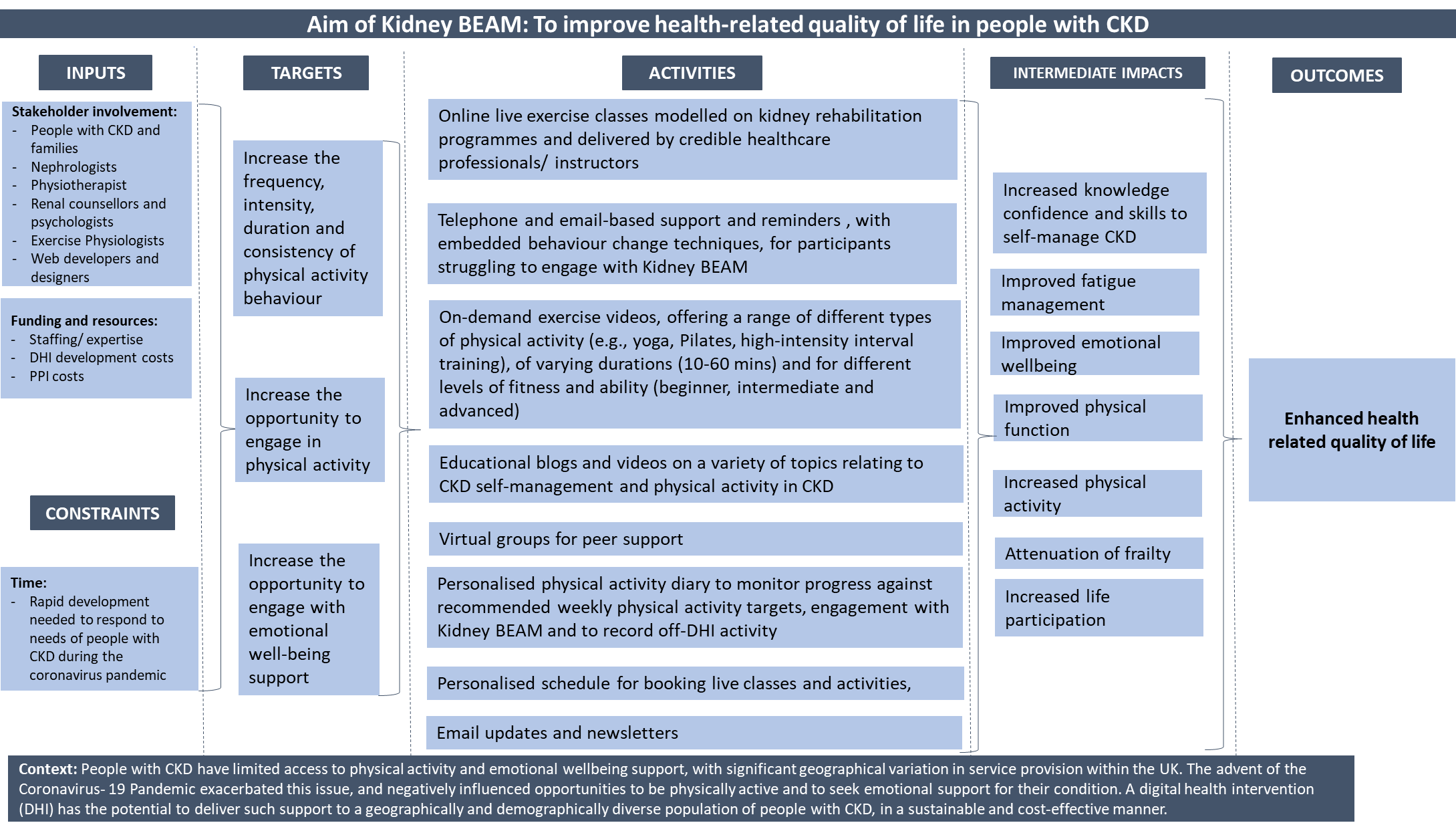


Figure S1. Logic model for Kidney BEAM.

1. Design of the Kidney BEAM DHI

Having identified the key functions of the Kidney BEAM programme and selected appropriate BCTs, a creative design phase began. This combined participatory co-design^4^ with user-centred agile software design^16^ to rapidly create an intuitive and engaging DHI, which had a high potential for effectiveness.

With the support and input of user experience (UX) designers, the stakeholder group generated ideas and made decisions regarding how the intervention functions could best be operationalised and delivered via a DHI. A user-centred agile design approach was selected to allow for rapid high-quality development, whilst also integrating the needs of end-users throughout.^16^ To this end, extensive up-front design was avoided, and instead, functionality was developed incrementally, whilst tangible ‘mock-ups’ and wireframes were initially used to gather feedback and iteratively design Kidney BEAM. These short incremental and iterative feedback cycles were used to make ongoing enhancements, progressing early prototypes towards a minimally viable product ready for more extensive user testing.^16^ Parallel design and development tracks were used, with design activities starting one sprint ahead of development activities.^16^

Throughout the design phase, the needs of those with low literacy (including health and digital literacy) were considered and integrated to enhance accessibility. This resulted in the creation of ‘how-to guides’ as well as the provision of supplementary telephone and in-person support, for people requiring technical help. A partnership was formed with the digital inclusion charity Citizens Online^17^ to provide skills training and support access to Kidney BEAM. The charity also supported people to use Microsoft ‘Edge Translate’, a digital translation tool for online resources. Written content was checked for readability and the use of lay language throughout and was made available in large font.

#### User testing phase

At the end of the four-week initial development phase, there was a six-month user testing phase to assess how engaging the intervention was, and direct further refinement. This phase is reported in detail elsewhere,^18^ but briefly, between 1 June 2020 and 30 November 2020 all people in the UK living with CKD aged ≥18 years were eligible to sign up. They completed a voluntary electronic survey on sign up which was repeated at six months, to establish whether they met current PA guidelines, investigate perceptions of health, and to collect usability and acceptability data. There were 959 sign-ups to Kidney BEAM in the 6 months. A pre-post survey revealed a 67% increase in people using Kidney BEAM meeting current national physical activity guidance of 150 minutes of moderately intense physical activity per week, a 20% increase in people reporting 75 mins or more of vigorous activity per week and 74% and 67% increase in number people meeting the twice-weekly strength training,^19^ and who perceived their energy levels to be good or very good, respectively. More than 1.9,000 movement classes were completed during the 6 months, with a total of 60,000 movement minutes. Refinements included the addition of video content suitable for less mobile individuals (delivered in sitting).

The development phase ended after the six-months, to allow Kidney BEAM to be rapidly available to people with CKD during the COVID-19 pandemic and to commence formal evaluation as part of the Kidney BEAM trial.^20^

**References**

1. O'Cathain A, Croot L, Duncan E, et al. Guidance on how to develop complex interventions to improve health and healthcare. *BMJ open* 2019;9(8):e029954.

2. West R, Michie S. A guide to development and evaluation of digital behaviour interventions in healthcare: Silverback Publishing 2016.

3. NIHR. Guidance on co-producing a research project 2021 [Available from: <https://www.learningforinvolvement.org.uk/?opportunity=nihr-guidance-on-co-producing-a-research-project>.

4. Clemensen J, Larsen SB, Kyng M, et al. Participatory design in health sciences: using cooperative experimental methods in developing health services and computer technology. *Qualitative health research* 2007;17(1):122-30.

5. Zeng J, Bennett PN, Hill K, et al. The exercise perceptions of people treated with peritoneal dialysis. *Journal of Renal Care* 2020;46(2):106-14.

6. Barriers and facilitators for engagement and implementation of exercise in end‐stage kidney disease: future theory‐based interventions using the behavior change wheel. Seminars in dialysis; 2019. Wiley Online Library.

7. Clarke AL, Young HM, Hull KL, et al. Motivations and barriers to exercise in chronic kidney disease: a qualitative study. *Nephrology Dialysis Transplantation* 2015;30(11):1885-92.

8. Castle EM, Greenwood J, Chilcot J, et al. Usability and experience testing to refine an online intervention to prevent weight gain in new kidney transplant recipients. *British Journal of Health Psychology* 2021;26(1):232-55.

9. Billany RE, Smith AC, Stevinson C, et al. Perceived barriers and facilitators to exercise in kidney transplant recipients: A qualitative study. *Health Expectations* 2022;25(2):764-74.

10. Michie S, Atkins L, West R. The behaviour change wheel. *A guide to designing interventions 1st ed Great Britain: Silverback Publishing* 2014;1003:1010.

11. Michie S, Van Stralen MM, West R. The behaviour change wheel: a new method for characterising and designing behaviour change interventions. *Implementation science* 2011;6(1):1-12.

12. England N. A digital solution to provide physical and emotional wellbeing for renal patients across the UK 2022 [Available from: <https://transform.england.nhs.uk/key-tools-and-info/digital-playbooks/renal-digital-playbook/a-digital-solution-to-provide-physical-and-emotional-wellbeing-for-renal-patients-across-the-uk/> accessed 11th April 2023.

13. McKane La. Renal Medicine GIRFT Programme National Specialty Report 2021 [Available from: <https://gettingitrightfirsttime.co.uk/wp-content/uploads/2021/09/Renal-Medicine-Sept21k.pdf> accessed March 2023.

14. Jenkins K. NHS England & Improvement Renal Services Transformation programme: MA Healthcare London, 2021:160-61.

15. Michie S, Richardson M, Johnston M, et al. The behavior change technique taxonomy (v1) of 93 hierarchically clustered techniques: building an international consensus for the reporting of behavior change interventions. *Annals of behavioral medicine* 2013;46(1):81-95.

16. Brhel M, Meth H, Maedche A, et al. Exploring principles of user-centered agile software development: A literature review. *Information and software technology* 2015;61:163-81.

17. Online C. Citizens Online 2023 [Available from: <https://www.citizensonline.org.uk/> accessed March 2023.

18. Mayes J, Billany RE, Vadaszy N, et al. The rapid development of a novel kidney-specific digital intervention for self-management of physical activity and emotional well-being during the COVID-19 pandemic and beyond: Kidney Beam. *Clinical kidney journal* 2022;15(3):571-73.

19. Baker LA, March DS, Wilkinson TJ, et al. Clinical practice guideline exercise and lifestyle in chronic kidney disease. *BMC nephrology* 2022;23(1):1-36.

20. C.G Walklin HMLY, E Asghari, S Bhandari, R.E Billany, N Bishop, K Bramham, J Briggs, J.O Burton, J Campbell, E.M Castle, J Chilcot, N Cooper, V Deelchand, M.P.M Graham-Brown, A Hamilton, M Jesky, P.A Kalra, P Koufaki, K Macafferty, A.C Nixon, H Noble, Z.L Saynor, C Sothinathan, M.W Taal, J Tollitt, D Wheeler, T J Wilkinson, J Macdonald, S.A Greenwood. The effect of a novel, digital physical activity and emotional well-being intervention on health-related quality of life in people with chronic kidney disease: trial design and baseline data from a multicentre prospective, wait-list randomised controlled trial (Kidney BEAM). *[submitted]* 2023
