# Supplementary material for: The development and internal pilot trial of a digital physical activity and emotional well-being intervention (Kidney BEAM) for people with chronic kidney disease
Source: Sci Rep. 2024 Jan 6;14:700. doi: 10.1038/s41598-023-50507-4 (PMC10771473; doi:10.1038/s41598-023-50507-4)
Supplement: Supplementary file 2 — Supplementary Information 2. [file 41598_2023_50507_MOESM2_ESM.docx]

Supplementary material 2. Topic guide for the Kidney BEAM pilot trial

| **Intervention related topics** | - What were your impressions of Kidney BEAM when you first logged on? |
| --- | --- |
|  | - Did you join any of the groups on Kidney BEAM? |
|  | - Did you read any of the blog posts? |
|  | - One section of the site offers short educational videos on a variety of topics such as falls, goal setting and the benefits of physical activity. Did you watch any of these? |
|  | - Another section offers exercise classes ‘on demand’. Did you try any of these? |
|  | - Did you try any ‘live classes? |
|  | - How often did you do an exercise programme per week? |
|  | - Did you upload any ‘offline’ physical activity into the diary on Kidney BEAM? |
|  | - What support was available to help you be more physically active? |
|  | - Was there anything that put you off using Kidney BEAM? |
|  | - How do you think we could improve Kidney BEAM in the future? |
|  | - How did you hear about Kidney BEAM? |
| **Trial related topics** | - What made you decide to take part in the Kidney BEAM trial? |
|  | - How could we get more people to participate? |
|  | - Could you tell me about the process of signing up to this study? |
|  | - As part of this study there was a 50/50 chance of being put into the group which had access to Kidney BEAM immediately, or the other group who had to wait 12 weeks. How did you feel about this? |
|  | - As part of the study, you were asked to complete some assessments or tests to help the trial researchers understand if Kidney BEAM is effective or not. |
|  | - What did you think about doing these assessments online/ over the phone? |
|  | - What would help other people taking part complete these assessments/ tests? |
|  | - Some people may not manage to complete a research study like the one you have taken part in, which may happen for several reasons [*give examples as needed*]. |
|  | - What would you like to happen once the study is completed? |
