# Supplementary material for: The development and internal pilot trial of a digital physical activity and emotional well-being intervention (Kidney BEAM) for people with chronic kidney disease
Source: Sci Rep. 2024 Jan 6;14:700. doi: 10.1038/s41598-023-50507-4 (PMC10771473; doi:10.1038/s41598-023-50507-4)
Supplement: Supplementary file 3 — Supplementary Information 3. [file 41598_2023_50507_MOESM3_ESM.docx]

Supplementary material 3. Demographics of those who declined to participate.

|  | | ***N*=166** |
| --- | --- | --- |
| **Age** (years) |  | 58±15 |
| **Sex** *n* (%) | Female | 54 (33%) |
|  | Male | 112 (67%) |
| **Ethnicity** *n* (%) | White British | 113 (68%) |
|  | Asian or Asian British | 27 (16%) |
|  | Black or Black British | 16 (10%) |
|  | Other ethnic background | 3 (2%) |
|  | Information not provided | 7 (4%) |
| **CKD stage** *n* (%) | Stages 1-5 (not receiving renal replacement therapy) | 41 (25%) |
|  | Haemodialysis | 27 (16%) |
|  | Peritoneal dialysis | 27 (16%) |
|  | Transplant | 71 (43%) |
