# Supplementary material for: The development and internal pilot trial of a digital physical activity and emotional well-being intervention (Kidney BEAM) for people with chronic kidney disease
Source: Sci Rep. 2024 Jan 6;14:700. doi: 10.1038/s41598-023-50507-4 (PMC10771473; doi:10.1038/s41598-023-50507-4)
Supplement: Supplementary file 4 — Supplementary Information 4. [file 41598_2023_50507_MOESM4_ESM.docx]

Supplementary Material 4. Prioritisation of suggested amendments to Kidney BEAM prioritised according to MoSCoW

|  | **MUST HAVE** | **SHOULD HAVE** | **COULD HAVE** | **WILL NOT HAVE** |  |
| --- | --- | --- | --- | --- | --- |
| **Suggested amendment** | **Necessary** | **Important** | **Beneficial** | **Not a priority** | **Rationale** |
| Provide more CKD stage and life stage specific content |  |  |  |  | Packages of CKD stage specific information added to kidney BEAM |
| Provide more detailed information for those who are not newly diagnosed. |  |  |  |  | Useful to have for participants who have had CKD for a longer-period of time, but will not influence engagement with the physical activity components of the intervention |
| Enhance ease of navigation via support and ‘how to videos’ |  |  |  |  | Essential to increase usability and simple to create and embed within the DHI on- boarding process |
| Highlight new content on the site |  |  |  |  | Provided as part of the telephone support and on-boarding process, but future adaptations may be made to support future implementation |
| Provide advice on the key aspects of Kidney BEAM to engage with |  |  |  |  | Provided as part of the telephone support and on-boarding process, but future adaptations may be made to support future implementation |
| Offer shorter physical activity options |  |  |  |  | Currently cost prohibitive but likely to be important for future implementation |
| Offer a more extensive live class timetable |  |  |  |  | Currently cost prohibitive but likely to be important for future implementation |
| Allow more leeway with live class bookings |  |  |  |  | Unable to change this as it is a safety requirement for the classes |
| Mandate more weekly engagement with Kidney BEAM |  |  |  |  | Would change the minimum amount of activity currently mandated within the trial protocol, but may be important for future implementation |
| Create text message reminders about booked classes |  |  |  |  | Functionality prohibitive and may be demotivating to some participants |
| Allow physical activity diary to be synced with wearable devices and provide personal summary analytics |  |  |  |  | Currently cost and functionality prohibitive but likely to be important for future implementation |
