# Supplementary material for: The development and internal pilot trial of a digital physical activity and emotional well-being intervention (Kidney BEAM) for people with chronic kidney disease
Source: Sci Rep. 2024 Jan 6;14:700. doi: 10.1038/s41598-023-50507-4 (PMC10771473; doi:10.1038/s41598-023-50507-4)
Supplement: Supplementary file 5 — Supplementary Information 5. [file 41598_2023_50507_MOESM5_ESM.docx]

Supplementary material 5: Script to support recruitment to the Kidney BEAM study.

**Script for the BEAM study**

1. Check you have right person on the phone
2. “ Hi, my name is [name], I’m calling from [site]. It’s nothing at all to worry about. I’m calling because [routine healthcare professional name here] thinks you might be interested in a study we are running, and I wonder if I might be able to tell you a little bit about it?”

If they agree...

3.“We know that being more physically active is beneficial for people living with kidney disease. It helps to keep your blood pressure down, which can protect your kidneys from damage, and it can help keep you strong and independent, which is important for people with kidney disease because it can affect your muscles. It can also help boost your energy levels and help with symptoms like tiredness” {can tailor this to CKD stage]

4. “unfortunately we also know that not many people with kidney disease have access to any kind of help to support them to be more active. Because of this, we have developed a really lovely and unique online platform called Kidney BEAM which is especially designed for people with kidney disease.”

5. “This platform gives you access to live and on-demand movement classes which are all led by specialist physiotherapists, or trained instructors who themselves are living with kidney disease.

6. “There is something on there for all levels of ability, so even if you haven’t been active recently, or you are new to it, there will be something for you. The classes have a seated and a standing option so you can pick what feels right to you.”

7. “There is also a range of different types of classes to try on demand, so if you have ever wanted to try Pilates, yoga, HIT or strength training you will find something to suit you.”

8.”The programme lasts for 12 weeks, and we hope people will use it twice a week. All the activity you do outside of the platform also counts towards this. You can do the classes live with the physio, or on-demand at any time which fits into your schedule.”

6. “We are asking you to take part in this study because we want to see if this platform can help to improve people’s mental and physical health”

7. “It’s up to you if you decide to take part or not. If you don’t want to then it won’t affect any of the care you receive. Similarly, if you decide to take part and later decide to drop out, that’s completely fine.”

8. “If you do decide to take part I will ask for your email and send you a link to an online consent form. Once you fill this in you will have a 50/50 chance of either being in a group that has access to the platform right away, or one that waits 12 weeks and then has access. This is done randomly just so we can compare the effects of using the platform with not using it, but both groups will have access in the end. Being put into the group which has to wait doesn’t mean that you aren’t suitable to be more active.”

7.”You will be asked to take part in a brief assessment at the start of the study and at the end. These assessments are done online and will be arranged at your convenience, so there is no need to travel anywhere. We will ask you to fill in seven questionnaires and to do a sit-stand test. This involves seeing how many times you can stand from a chair in 60 seconds and tells us about your strength. We will also ask for your permission to look in your medical records and to look at recent blood tests you may have had. We won’t ask you to have any additional blood tests. “

8. “We are also asking some participants to take part in an interview online or over the phone. This is just to find out what you thought of the platform and how we might improve it in the future. The interview will be audio recorded and everything you say will be anonymised. If we use any quotes from you, you won’t be personally identifiable. The interview is also optional, so you don’t have to take part in this if you don’t want to. “

9. “The benefits of taking part are that it might help you to feel better, to improve your fitness, strength, and physical health. You will also be helping to improve care for future generations of people with kidney disease.” [can be tailored to the participants aims]

10. “The potential risks of taking part are the same with any type of activity programme. There is a small risk of injury – for example pulling a muscle. We have tried to reduce this as much as possible by ensuring the classes are run by trained specialist physiotherapists and ensuring that you have an assessment beforehand. We have also checked with your doctor that you are suitable to take part beforehand.”

11. “Do you have any questions for me? What do you think? Would it be ok for me to send some written information out to you by email? This just outlines what I have talked through today.”

12. If yes – collect and record email on spreadsheet. Send them out the PIS with the template email. Explain that you will call them back in a few days to see if they have any questions and to see whether or not they would like to take part.

13. If no – thank them for their time
